# An ancient testis-specific IQ motif-containing H gene regulates specific transcript isoform expression during spermatogenesis

**DOI:** 10.1242/dev.201334

**Published:** 2023-04-04

**Authors:** Paula Navarrete-López, Marta Lombó, Maria Maroto, Eva Pericuesta, Raúl Fernández-González, Priscila Ramos-Ibeas, María Teresa Parra, Alberto Viera, José Ángel Suja, Alfonso Gutiérrez-Adán

**Affiliations:** ^1^ Departamento de Reproducción Animal, INIA-CSIC, Avenida Puerta de Hierro 12. Local 10, 28040 Madrid, Spain; ^2^ Unidad de Biología Celular, Departamento de Biología, Facultad de Ciencias, Universidad Autónoma de Madrid, 28049 Madrid, Spain

**Keywords:** *Iqch*, Spermatogenesis, Transcript variants, lncRNAs, Splicing regulation

## Abstract

Spermatogenic cells express more alternatively spliced RNAs than most whole tissues; however, the regulation of these events remains unclear. Here, we have characterized the function of a testis-specific IQ motif-containing H gene (*Iqch*) using a mutant mouse model. We found that *Iqch* is essential for the specific expression of RNA isoforms during spermatogenesis. Using immunohistochemistry of the testis, we noted that *Iqch* was expressed mainly in the nucleus of spermatocyte and spermatid, where IQCH appeared juxtaposed with SRRM2 and ERSP1 in the nuclear speckles, suggesting that interactions among these proteins regulate alternative splicing (AS). Using RNA-seq, we found that mutant *Iqch* produces alterations in gene expression, including the clear downregulation of testis-specific lncRNAs and protein-coding genes at the spermatid stage, and AS modifications – principally increased intron retention – resulting in complete male infertility. Interestingly, we identified previously unreported spliced transcripts in the wild-type testis, while mutant *Iqch* modified the expression and use of hundreds of RNA isoforms, favouring the expression of the canonical form. This suggests that *Iqch* is part of a splicing control mechanism, which is essential in germ cell biology.

## Teaser

**Summary:** *Iqch* mutant mice reveal that *Iqch* controls a network of alternative splicing events in postmitotic germ cells that are necessary to establish the correct protein isoforms required for spermatid development.

## INTRODUCTION

Spermatogenesis is a complex process involving the somatic proliferation of spermatogonia, meiotic divisions that give rise to haploid cells, and postmeiotic differentiation of spermatids or spermiogenesis, which finally leads to the production of mature spermatozoa. The testis is a very heterogeneous organ composed of different stages or cell types where there is continuous production of male gametes (Griswold, 2016). First, spermatogonial stem cells go through a series of mitotic divisions and differentiation processes giving rise to undifferentiated and then differentiated spermatogonia producing preleptotene spermatocytes. Subsequently, meiosis I, which involves a prolonged prophase, comprises the substages leptotene, zygotene, pachytene, diplotene and diakinesis. During these steps, recombination, homologous chromosome pairing and synapsis take place. Meiosis I is then followed by the second meiotic division, when homologous chromosomes separate and give rise to haploid round spermatids. The third process is called spermiogenesis, when round spermatids go through several differentiation stages to become elongated spermatids and finally sperm. These events include dramatic changes, such as loss of most of the cytoplasm, acquisition of a flagellum, acrosome formation, reorganization of mitochondria, nuclear elongation and chromatin compaction by replacement of most histones. The last is mediated first by transition proteins and then by protamines, and this replacement results in transcriptional silencing during later spermiogenesis stages (Geisinger, 2008). The process of germ cell differentiation is sustained by the surrounding somatic cells, mainly Sertoli and Leydig cells, but also myoid cells, endothelial cells, macrophages, innate lymphoid cells and mesenchymal cells. This multistage process requires tight regulation of gene expression and is still not fully understood (Massart et al., 2012). Multidisciplinary efforts are needed to gain insight into the mechanisms that drive spermatogenesis and that could affect male fertility. The identification of genes that are crucial to the process of sperm production is necessary to further our understanding of the transcriptional program of spermatogenesis. Several thousands of testis-enriched genes (Lu et al., 2019) exist, and some of these have been identified as essential for the maintenance of the fine-tuned mechanisms needed to produce fertile spermatozoa (Greenbaum et al., 2006; Singh and Rajender, 2015; Castaneda et al., 2017; Wu et al., 2020).

In the search for currently unknown regulatory networks, we here examine the functions of IQ domain-containing H gene (*Iqch*). This testis-specific gene, which has been identified in humans and mice, is conserved across all mammalian species analysed so far, and is also found in chicken, zebrafish and frog (https://www.ncbi.nlm.nih.gov/homologene/11258). IQCH is a long protein (1071 amino acids in mice) containing one IQ motif [a short calmodulin (CaM)-binding motif containing conserved Ile and Gln residues], which is responsible for CaM binding in the absence of calcium. Calcium is known to participate in many biological processes during spermatogenesis (Darszon et al., 2011), and abnormal calcium homeostasis has been associated with impaired spermatogenesis (Chiarella et al., 2004; Shambharkar et al., 2015). IQCH also contains a carbamate kinase-like domain, a Zn-dependent exopeptidase domain and a lactate dehydrogenase (LDH) C-terminal-like domain. By analysing genomic regions associated with inbreeding-related reductions in semen quality traits in a cattle population, *Iqch* has been identified as a potential gene for determining male fertility and the percentages of live spermatozoa (Ferenčaković et al., 2017).

In the present study, we examine the impacts of *Iqch* on male fertility, testis phenotype and gene expression throughout the process of spermatogenesis. In *Iqch* mutant mice that were generated, males were found to be infertile but no effects were detected in females. Inactivation of *Iqch* in mice led to spermatogenesis abnormalities, low sperm production, and sperm function and morphology defects. Moreover, we noted that *Iqch* plays a regulatory role in spermatogenesis, affecting the expression of protein-coding genes and lncRNAs, and controlling the expression of spermatogenesis-specific RNA isoforms.

## RESULTS

### Spatial and temporal patterns of IQCH during spermatogenesis

It has been reported in mice that IQCH is almost exclusively expressed in all cells within the seminiferous tubules of the testis, but with strong signals detected in spermatocytes and spermatids, suggesting it may play a regulatory role in testis development (Yin et al. 2005). To examine the location of the protein, IQCH was immunolocalized through acrosome staining. IQCH was detected in all cell types of the seminiferous tubules, and also in Leydig cells, but its location varied between nucleus or cytoplasm depending on the cell type (Fig. S1). In primary spermatocyte and Leydig cells, its location was mainly cytoplasmic with a few small and low-intensity spots visible in the nucleus (Fig. S1A-E). Cytoplasmic staining was still observed in the secondary spermatocyte, with the dotted nuclear signal predominating (Fig. S1F-J). In round spermatids, staining was exclusively nuclear, appearing as dots distributed throughout the nucleus (Fig. S1K-O). In elongated spermatids and immature spermatozoa with a large amount of cytoplasm still attached to the tail, only cytoplasmic staining was observed (Fig. S1P-T).

### *Iqch* is essential for murine spermiogenesis

**Fig. 1.**
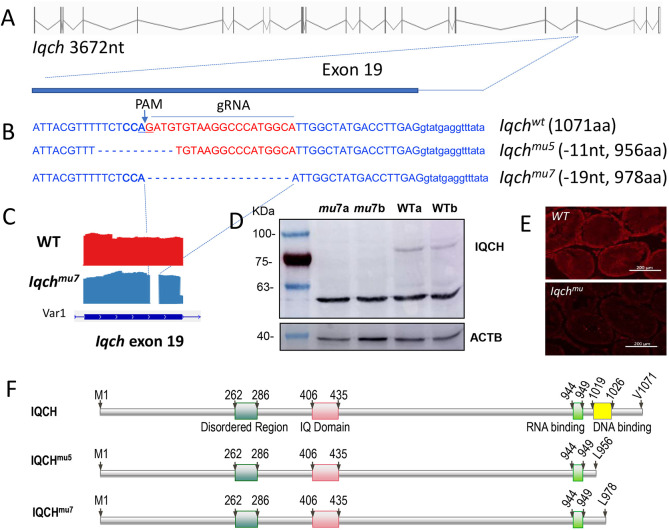
**Diagrams of *Iqch* gene structure and mutations produced by CRISPR/Cas9 in edited mice.** (A) The *Iqch* gene consists of 22 exons (vertical lines). (B) Nucleotide deletions produced by CRISPR/Cas9 in the edited mouse lines *Iqch^mu5^* and *Iqch^mu7^*. The number of nucleotides deleted and amino acids encoded by the wild-type and mutant proteins are indicated. Red letters indicate the guide RNA (gRNA). PAM sequences are indicated in bold blue. (C) RNA-seq coverage plot of the *Iqch* gene representing the wild-type data (red) and the *Iqch^mu7^* data (blue). The upper panel shows the detail of exon 19 of *Iqch*, showing the coverage and deletion in the mutant. The lower panel shows the size of exon 19 in the two principal variants of *Iqch*. (D) Western blot analysis of IQCH protein expression in the testes of two wild-type and two *Iqch^mu^* mutant mice, using ACTB as a loading control. The positions of molecular mass markers are indicated. (E) Immunolocalization of IQCH in cross-sections of seminiferous tubules of wild-type and *Iqch^mu^* mice (anti-IQCH in red). (F) Graphic diagrams of IQCH protein of wild-type and mutant mouse lines showing the structure of the main predicted domains.

To determine the role of *Iqch* during mammalian spermatogenesis, we used CRISPR-Cas9 technology to produce *Iqch* mutant mice with deletion of exon 19 (Fig. 1A). From the edited mice generated, two lines were selected with deletions of 11 and 19 nucleotides (*Iqch^mu5^* and *Iqch^mu7^*, respectively) that gave rise to expected proteins of 956 and 978 amino acids instead of the wild-type protein consisting of 1071 amino acids (Fig. 1B,F). As the two lines showed similar phenotypes, the following phenotyping results refer to the *Iqch^mu7^* line. In addition, as the mutant lines showed no fertility phenotype in heterozygosis, all analyses were performed in homozygous animals. The deletion carried by the edited line was confirmed by a Sashimi plot of the RNA-seq coverage of the *Iqch* gene exon 19 in wild-type and *Iqch^mu^* mice (Fig. 1C). Additionally, Western blotting revealed that the wild-type 1071 amino acid protein was absent in the testes of mutant mice (Fig. 1D). Interestingly, an unspecific band of about 50 kDa appeared, which could be due to some isoform that remained in the wild type and the mutant. Immunofluorescence analysis confirmed the absence of IQCH in mutant mice (Fig. 1E). The IQ domain (406-435) and the predicted RNA binding domain (944-949) were conserved in both IQCH mutants 5 and 7, while the predicted DNA binding domain (1019-1026) was lost in the truncated proteins (Fig. 1F). These differences at amino acid sequence reflect a change in predicted RNA-binding domain folding in the mutants (Fig. S2).

**Fig. 2.**
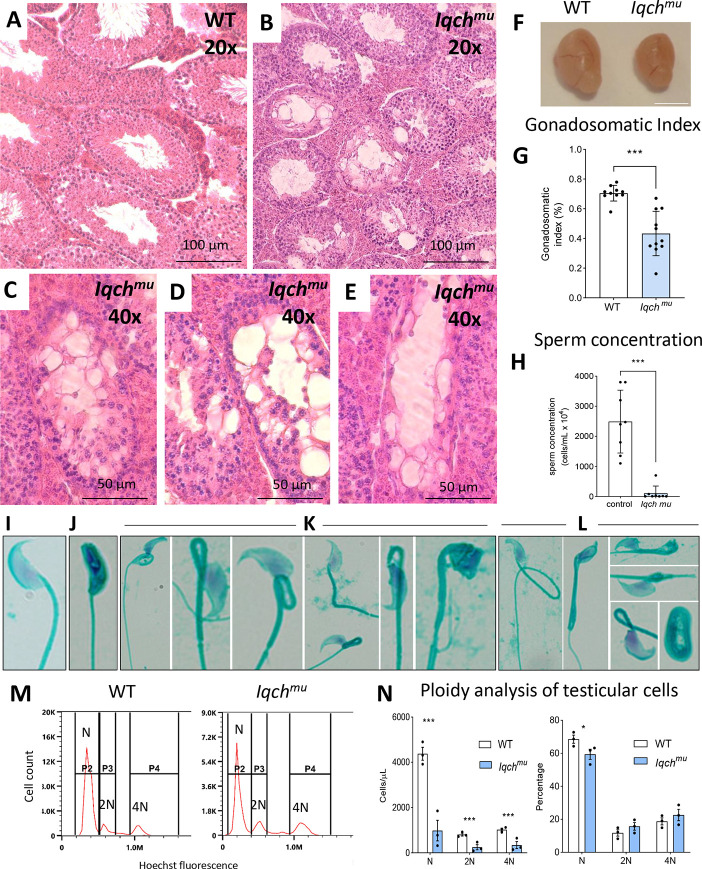
**The infertile phenotype of *Iqch^mu^* mice.** Hematoxylin and Eosin staining of testes of wild-type (A) and *Iqch^mu^* (B) mice. Scale bars: 100 μm. (C-E) Testis sections of *Iqch^mu^* mice showing abnormal individual seminiferous tubules. Scale bars: 50 μm. (F) Representative images of whole testis from wild-type and *Iqch^mu^* 4-month-old mice. Scale bar: 5 mm. (G) Wild-type (white, *n*=10) and *Iqch^mu^* (blue, *n*=11) testes sizes are shown as percentages of total body weight in 4-month-old mice. (H) Sperm concentration in wild type (white, *n*=8) and *Iqch^mu^* (blue, *n*=8). (I-L) Examples of normal (I) and abnormal (J-L) sperm isolated from the cauda epididymis in Iqchmu mice (stained with Spermac Stain Kit and observed under a standard bright-field microscope). Over 80% of sperm show some type of abnormality, including neck and midpiece defects (K,L), head defects (J-L) and tail defects (L). (M) Representative DNA ploidy histograms stained with Hoechst 33342 for wild-type and *Iqch^mu^* testicular germ cells. N, haploid germ cells; 2N, diploid cells (spermatogonia); 4N, tetraploid cells (germ cells in meiosis I and cells in G2/M-phase). (N) Distribution of cells according to DNA amount in wild-type and *Iqch^mu^* testis of adult mice (*n*=3/group): on the left, the number of testicular cells per μl present in each mouse; on the right, the proportion of cells. Data in G, H and N are mean±s.e.m. Statistical analyses were carried out using one-way ANOVA with a multiple comparison Tukey's test; *P<0.05, ***P<0.001.

Male and female edited mice developed normally, and, whereas females were fertile, males homozygous for the mutation were infertile. Although the *Iqch* mutant males copulated normally, they were completely infertile (20 males crossed with two females each were analysed). Histological observation of *Iqch^mu^* testis revealed some near-empty seminiferous tubules, disorganized tubules with abnormal spermatids and few spermatozoa (Fig. 2A-E; Fig. S3). The testes of adult mutant mice were smaller than wild type (Fig. 2F,G); sperm concentration was also reduced (Fig. 2H). The consequence of these would be azoospermia and male infertility. To analyse this phenotype, we analyse the chromosome segregation during meiosis. *Iqch^mu^* spermatocytes adequately progressed throughout meiosis, and they successfully achieved the processes of homologous chromosomes synapsis, recombination and segregation during both meiotic divisions (Figs S4 and S5).

To explore the nature of these spermatid defects, the outer acrosomal membrane was immunostained with FICT-conjugated PNA. Whereas a single uniform acrosomal structure was present in wild-type spermatids, fewer acrosomal structures were observed in *Iqch^mu^* spermatids (Fig. S3) and acrosomal cap structures were discontinuous, abnormally fragmented and with malformations (Fig. S6). In addition, sperm from mutant mice showed reduced viability, motility and increased sperm morphological abnormalities in the neck, midpiece, head and tail (Fig. 2I-L, Fig. S7 and Movies 1, 2).

To identify the stage of spermatogenesis that is being affected, we analysed the proportions of the different germ cells with 4n, 2n and n sets of chromosomes by using flow cytometry (Fig. 2M,N). In the mutant testis, there is a reduction of all the cell types (Fig. 2M,N, left; in Fig. 2M, *y* axis shows different scale in wild-type and *Iqch* testicles), but when we analysed the proportions we observed a significant reduction in only the population of haploid cells, i.e. round and/or elongated spermatids (Fig. 2N right), but the cell populations of 2n and 4n are not affected.

### The *Iqch* mutation gives rise to a considerably modified transcriptome profile of mouse testis

To establish the molecular basis for the spermatogenesis defects observed in *Iqch* mutant mice, we performed RNA-seq analysis on testes of four wild-type and four *Iqch^mu^* adult mice. After the mapping step, around ∼34 million paired-end reads were successfully assigned per sample, with an alignment rate >96% in all cases (Table S1A). Through gene quantification, an average of ∼24,060 genes were detected among samples. One *Iqch* mutant sample, which was considered an outlier, was excluded from further analysis after normalization. Finally, both experimental groups were clustered together according to the gene expression profiles of the top significant differentially expressed genes (DEGs) (Fig. S8).

**Fig. 3.**
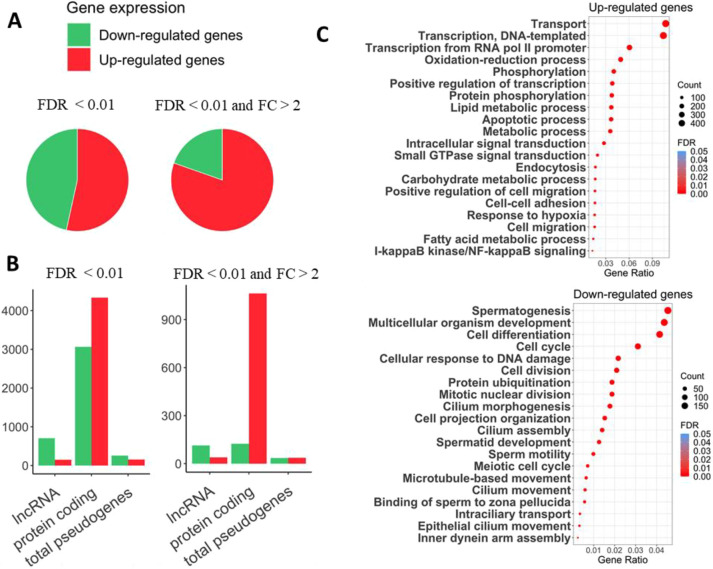
**RNAseq differential expression analysis.** (A) Pie charts showing proportions of upregulated and downregulated genes for the set of DEGs with FDR<0.01 and the DEGs with FDR<0.01 and fold changes>2. (B) Bar plot of the number of genes belonging to the three main gene types in the set of DEGs with FDR<0.01, FDR<0.01 and FC>2. (C) Dot plots of the top significant GO-BP terms in which the upregulated and downregulated gene sets are enriched, sorted by the gene ratio that represents the number of DEGs involved in the biological process over the total number of DEGs in the set. The size of the dots reflects the gene count that contributes to the enrichment of the term, while their colour represents the FDR value of the over-representation analysis.

Our differential gene expression analysis of the *Iqch* mutant and control groups (common results in edgeR and DEseq2 analysis with FDR<0.01) revealed the upregulation of 4657 genes and the downregulation of 4057 genes in *Iqch* mutant testes. Overall, 8714 genes were differentially expressed in the wild-type and mutant tissue samples. Of these, 1423 DEGs showed a fold change above 2, the vast majority of them, upregulated (Fig. 3A, Table S1B-F, Fig. S9). Thus, the absence of *Iqch* seems to induce widespread changes in the transcriptome profile of the testis, with the ultimate consequence of infertility. Notably, in the absence of *Iqch*, most genes showing high fold changes were upregulated (Fig. 3A), suggesting that under normal conditions, *Iqch* may be involved in repressing specific pathways that are essential for the maintenance of normal spermatogenesis.

When we considered only an FDR<0.01, the mainly affected gene types were 7395 protein-coding genes, followed by 852 long non-coding RNAs (lncRNAs) and, in third position, 402 pseudogenes (Fig. 3B, Fig. S10A). Interestingly, among the protein-coding genes, upregulated genes outnumbered downregulated genes, whereas lncRNAs were predominantly downregulated. Specifically, there were 705 underexpressed and 147 overexpressed lncRNAs in *Iqch* mutant mice. Moreover, when considering DEGs undergoing the greater fold changes, eight out of the top 10 were lncRNAs (Fig. S10B). This indicates the significant dysregulation of lncRNAs in *Iqch* mutant testis. Generally, lncRNAs show tissue- or cell-specific and time-dependent expression patterns, and have been found to be abundantly expressed in a testis-specific manner (Hong et al., 2018; Trovero et al., 2020; Li et al., 2021). In fact, 417 DE lncRNAs were restricted to the testis, and 125 were preferentially expressed in this tissue. These exert their function in a wide variety of regulatory processes, such as gene expression, chromatin organization, splicing, mRNA stability, translation or post-translational modifications (Marchese et al., 2017; Zhang et al., 2019), which are likely essential for the maintenance of normal spermatogenesis.

When analysing the over-representation of the set of DEGs in biological processes, we found enrichment in a variety of functions (Fig. 3C, Table S2). Upregulated genes were enriched in transport and in transcriptional regulation mechanisms involving transcription factors and other genes that participate in the activation of gene expression, i.e. *Spi1*, *Jun*, *Tcf3*, *Nfatc3* and *Nfatc4*, which may explain the upregulation of so many genes. Moreover, loss of *Iqch* function led to the increased expression of genes involved in some metabolic pathways, mainly lipid metabolism and, particularly, fatty acid β-oxidation, as well as oxidation-reduction and phosphorylation processes. For example, there was enrichment in upregulated genes that participate in the response to oxidative stress, such as *Camk2g*, *Idh1* and *Gpx3*. Oxidative damage is known to promote lipid peroxidation throughout spermatogenesis, which can ultimately modify sperm motility and fertilization capacity (Huyghe et al., 2006). In addition, some of the genes showing the greater significant difference, *Tmem176a*, *Tmem176b, Serpina3n* and *Serpina5* (Fig. 3B), were those associated or colocalized with the Golgi apparatus (Drujont et al., 2016; Tjondrokoesoemo et al., 2016).

Among the genes that emerged as downregulated, those involved in spermatogenesis and sperm-related functions such as cilium assembly, sperm motility, spermatid development, acrosome assembly and meiotic cell cycle, among others, were over-represented. Spermatogenesis failure in mutant mice was evidenced by the diminished expression of 184 spermatogenesis-associated genes, including *Hsf2bp*, *Cib1*, *Crem*, *Insl6*, *Tnp1*, *Ros1*, *Brdt* and some Tssk and Spata family genes. Other essential genes that were differentially expressed were *Mnd1*, *Meiob*, *Meioc*, *Aurka*, *Sycp1* and *Piwil1*, which are involved in the meiotic cell cycle, and *Dnah11*, *Dnah1* and the Catsper channel protein genes involved in sperm motility. In addition, *Iqch^mu^* mice featured lower *Iqch* RNA expression than wild-type (−3.4-fold), which was also seen at the protein level by western blotting, whereby the IQCH band disappeared in the mutants (Fig. 1D).

### Effect of *Iqch^mu^* on the RNA expression of different testis cell types

**Fig. 4.**
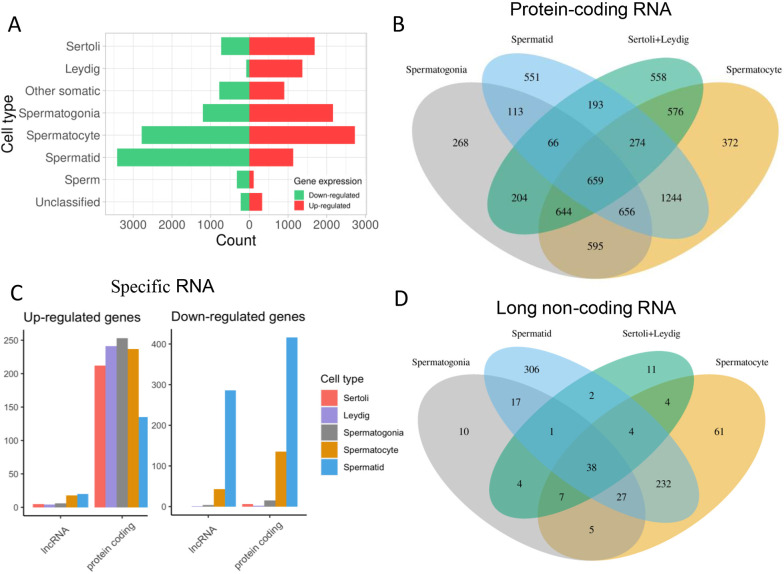
**Characterization of the cell types of the set of DEGs.** DEGs were classified based on the different studies that have characterized the stages of wild-type testis. (A) Bar plot showing the number of upregulated and downregulated DE genes in each cell type. (B) Venn diagram of DE protein-coding genes of the main testis cell types. Separate and overlapping expression between samples is shown. (C) Bar plots of the DE protein-coding and lncRNAs for each cell type. Bar plots to the left represent total RNA, while bar plots to the right represent cell-type specific RNA. (D) Venn diagram of DE lncRNAs of the four major cell types. Separate and overlapping expression between samples is shown.

Although *Iqch* is expressed in male germline cells spanning from spermatogonia to spermatids and in testis somatic cells, our DEG data from bulk RNA-seq of adult testis could not discriminate an effect of *Iqch^mu^* on the different cell types. To find out to which cell stage of the testis our detected DEGs belonged, we assigned genes to specific cell types based on a single-cell RNA-seq dataset (Green et al., 2018). Clustering of all cells served to identify four main cell types: spermatogonia, spermatocytes, spermatids and somatic cells (Fig. S11). When we integrated this analysis with our data, DEGs were assigned to the following cell types: 5511 spermatocyte genes, 4550 spermatid genes, 3364 spermatogonia genes, 2416 Sertoli cell genes, 1682 genes expressed in other somatic cells, 1453 Leydig cell genes, 445 sperm genes and 555 genes that remained unclassified (Table S3A). These data suggest that the *Iqch* mutation had a differential impact on the transcriptome of each testis cell type. Notably, the transcriptome of testicular somatic cells was also modified, as well as that of other somatic cells. Genes involved in early stages of spermatogenesis were more upregulated, as we found 2162 upregulated genes versus 1202 downregulated genes in spermatogonia, while gene expression at late stages was mainly reduced, with 3417 genes showing decreased expression versus 1133 with increased expression in spermatids (Fig. 4A). In Leydig cells, 1372 genes showed increased expression and only 81 were downregulated (Fig. 4A); when looking at genes showing a fold change over a threshold of two Leydig genes featured the greater dysregulation (Table S3A), although the total number of genes was the lowest among germ cells and somatic cells. The higher number of downregulated genes observed at the spermatid stage in the *Iqch* mutant testes could be related to the reduction of spermatid cells observed at this stage.

We then went on to analyse the genes that were differentially expressed in each specific cell type. Among the four predominant cell types in testis: spermatogonia, spermatocytes, spermatids and testicular somatic cells (Sertoli+Leydig cells), 707 DEGs were common to all cell types, and among them, 659 were protein-coding genes (Fig. 4B) and 38 lncRNAs (Fig. 4D). In summary, spermatid-specific genes were predominant over the rest of the cell types, including 551 protein-coding genes and 306 lncRNAs. Remarkably, lncRNAs were particularly altered and mainly downregulated in spermatids: in total 627; of these, 563 were underexpressed in *Iqch* mutants (Fig. 4C-D, Table S3A). Spermatids are known to have a lncRNA-specific expression pattern in the testis that may tightly regulate the process of spermiogenesis (Trovero et al., 2020) and that seemed to be impaired upon *Iqch* mutation, some of which can be due to the cell composition differences of spermatids.

The functional annotation of genes of the different cell types (Fig. S12, Table S4) revealed the presence of common upregulated processes among spermatogonia, spermatocytes, and Leydig and Sertoli cells, such as lipid metabolic, apoptotic, oxidation-reduction and oxidative stress response processes. Genes involved in transcription were especially upregulated in spermatogonia, spermatocytes and Sertoli cells, which could explain the abnormal transcriptome observed. In spermatogonia, mRNA processing and RNA splicing genes, including *Hspa8*, *Rbm22*, *Rbm17*, *Rbmx*, *Tsen34*, *Tsen2*, *Srsf6* and *Srsf2* were overexpressed. Cholesterol metabolism processes involving genes such as *Abca1*, *Apoe*, *Apoc1* and *Lrp1*, and glutathione metabolism processes involving *Nat8*, *Gpx3*, *Gstm1*, *Idh1* and *Gsta2* were modified in Leydig cells (Fig. S12A).

As previously mentioned, downregulated genes were mainly expressed in spermatid germ cells, and the mechanisms in which they participated were tightly linked to sperm development. The presence of mutant *Iqch* reduced the expression of genes participating in spermatogenesis in later stages, namely those driving sperm motility, cell differentiation, binding of sperm to zona pellucida, the cell cycle, the meiotic cell cycle, cilium morphogenesis, inner dynein arm assembly, acrosome assembly and intraciliary transport. (Fig. S12A,B). Therefore, the RNA-seq data indicates the reduced expression of spermatid-specific and spermiogenesis-associated genes, which agrees with the depletion of spermatid cells observed in the mutant tissue. Some of this gene dysregulation could be due to the reduction of the spermatid population in mutants. However, the expression of spermatid marker genes (*Tex21*, *Izumo1*, *Izumo2*, *Spata25*, *Akap4*, *Spag6*, *Tnp1*, *Tnp2*, *Prm1*, *Prm2*, *Prm3*, *H1f9*, *H1f7*, *H2ap*, *H2al2a*, *H2bl1* and *Hspa1l*) revealed that differences due to this are subtle, with a low fold-change in all cases.

In addition to protein-coding genes, we characterized lncRNAs by examining their interactions with proteins and mRNAs (Table S5). LncRNAs are important regulators of many processes based on their ability to orchestrate different molecular interactions. Altogether, 594 DE lncRNAs were annotated with interacting mRNAs and proteins: 4828 mRNAs and 3740 proteins. When we considered the 38 common lncRNAs that were dysregulated in spermatogonia, spermatocytes, spermatids and testicular somatic cells (Fig. 4D), 34 of them displayed interactions with 1250 proteins. We found that these common DE lncRNAs shared target proteins and, specifically, 77 proteins were seen to interact with three or more lncRNAs. The shared proteins were more abundant in dynamic processes, including sexual reproduction, chromatin remodelling and organelle organization (Fig. S13A). Interestingly, of the 588 DE lncRNAs showing interactions with proteins, 484 were downregulated and most of them were specific to or expressed mainly in the testis (282 specific to, 65 mainly expressed, 24 non-specific and 113 with no data available). In contrast, the 104 lncRNAs showing increased expression in the testis were mostly non-specific (46 non-specific, 20 specific, 13 mainly expressed and 25 with no data available) (Table S5). The same pattern was shown by lncRNAs that interacted with mRNAs (Table S5), suggesting that *Iqch^mu^* exerts a direct and testis-specific effect on downregulated lncRNAs but not on upregulated ones.

Additionally, we explored the interactions of DE spermatid lncRNAs that were the most abundant and relevant due to their specificity (Fig. 4D). Among the downregulated lncRNAs that in normal conditions are expressed in spermatids, 416 were found to show interactions with 3705 proteins. Interestingly, these target proteins were involved in the processes of the cell cycle, cell division, regulation of Rho protein signal transduction, activation of GTPase activity, phosphorylation, cell-cell adhesion, cilium morphogenesis, microtubule-based movement, mRNA processing and RNA splicing (Fig. S13B).

### *Iqch^mu^* exhibits differential splicing of RNAs

**Fig. 5.**
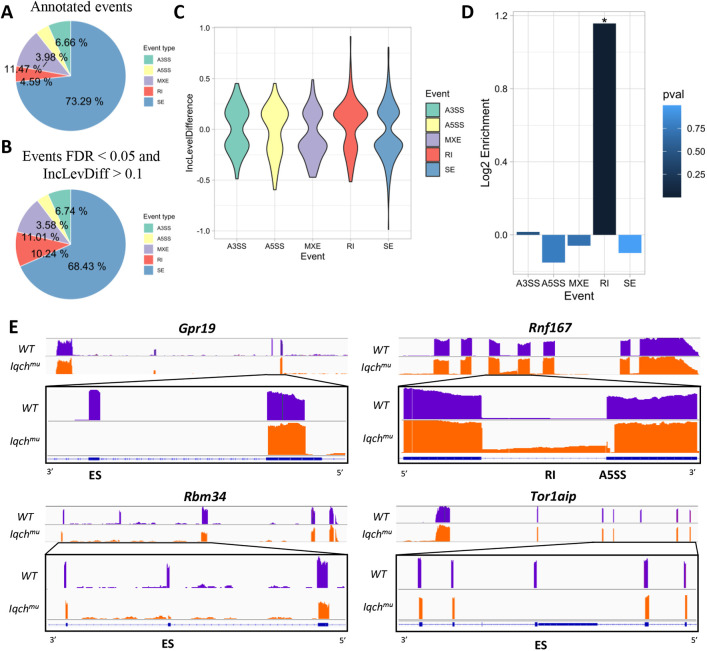
**Differential splicing event analysis by rMATS.** (A,B) Pie charts representing the proportion of the different events (A) in the annotated reference and (B) in the significant events in the comparison between Iqch^mu^ and control samples, with FDR<0.05 and IncLevDiff (inclusion level difference)>0.1. (C) IncLevDiff distribution of the significant splicing events of each category in the form of violin plots. (D) Log2 fold enrichment of the differential event types, indicating the significance of enrichment of the event type with asterisks and its hypergeometric *P*-value, with a blue colour gradient. (E) Splice junction visualization of SE events through Sashimi plots of *Gpr19*, *Rbm34* and *Tor1aip* genes, and IR and A5SS events of *Rnf167*. For each gene, a general view of the coverage calculated for wild type (violet) and *Iqch^mu^* (orange) is depicted in the top panel. The bottom panel shows a zoomed region of the alternative splicing event. Read counts are shown using an identical scale in all samples. The canonical isoform is shown in blue below the panel.

With the aim of detecting local alternative splicing (AS) events, these events were categorized using the rMATS pipeline (Shen and Rajender, 2014) into five different groups: skipped exon (SE), alternative 5′ splice site (A5SS), alternative 3′ splice site (A3SS), mutually exclusive exons (MXE) and retained intron (RI). Overall, 1172 differential splicing events were identified in *Iqch^mu^* testes: 533 upregulated and 639 downregulated (Table S6A). Differences were observed among all splicing event types, establishing an FDR threshold of 0.05 and an inclusion level difference of 0.1 (inclusion level refers to the number of reads assigned to the event where there is inclusion of the respective exon, so that the exon is present in the final processed transcript). Exon-skipping events were the most abundant type, with 802 events taking place in 601 genes (Fig. 5A-D). Greater dysregulation was observed in SE and RI events, with inclusion levels ranging from −0.986 to 0.809 in SE events and from −0.516 to 0.915 in RI events (Fig. 5C). Considering enrichment over the total number of detected events in our RNA (Fig. 5A), IR events were significantly over-represented (Fig. 5D), with 120 differential events and a hypergeometric *P*=4.102182e-16 (Fig. 5C). In line with this, there was a higher inclusion of IR events rather than a lower inclusion, with 80 upregulated IR events and 40 downregulated IR events. These results suggest that *Iqch* could have a role directly or indirectly in the RNA-splicing process.

Specific examples of splicing alteration in SE (*Gpr19*, *Rbm34* and *Tor1aip1*) and RI and A3SS (*Rnf167*) in *Iqch^mu^* were related to spermatogenesis (Fig. 5E, Table S6). Interestingly, G protein-coupled receptor 19 (*Gpr19*) is preferentially expressed in the central nervous system (CNS), where it modulates the circadian clock in the CNS (Yamaguchi et al., 2021), and in pachytene spermatocytes in the testis (Rossi et al., 2004). RNA-binding motif protein 34 (*Rbm34*) is also preferentially expressed in the testis, and although its function remains unclear, its RNA recognition motif (RRM) domains may participate in mRNA processing. RING finger protein 167 (*Rnf167*) regulates lysosome positioning and endocytic trafficking (Deshar et al., 2016). torsin 1a interacting protein 1 (*Tor1aip1*) contributes to sperm-specific chromatin distribution, modulates cellular remodelling during spermiogenesis, and is involved in dynamic microtubule changes related to manchette formation and flagella development (Serrano et al., 2017).

### *Iqch* mutants show differential expression of RNA isoform variants

To explore whether *Iqch* may control the expression of certain RNA isoforms in the testis, we compared isoforms expressed in *Iqch* mutants and wild-type mice. Given the presence of novel unannotated transcripts in the testis, and the diversity and specificity of alternative splicing across tissues (Wang et al., 2008; Trapnell et al., 2010), we used a method, StringTie, that integrates genome-guided and *de novo* assembly approaches (Pertea et al., 2015). The resulting set of known and novel transcripts comprised 170,274 transcripts based on GRCm39 reference annotation and the RNA-seq samples. Quantification gave rise to read coverage tables, including a total of 107,260 transcripts detected in at least one group used in our differential expression and switching analyses. Transcript counts obtained by StringTie were the inputs for two different isoform analyses: differential transcript expression (DTE) using edgeR and limma voom, and differential transcript usage (DTU) using isoformSwitchAnalyzeR (Vitting-Seerup et al., 2019). When examining DTE, individual transcript counts were tested independently from the total transcriptional profile of the gene, whereas, using DTU, we detected isoforms showing a switch in the ratio between all transcripts of a gene, i.e. a change in the relative expression of the isoforms of a gene. Both analyses revealed the presence of differential isoform variants in the *Iqch^mu^* testis.

**Fig. 6.**
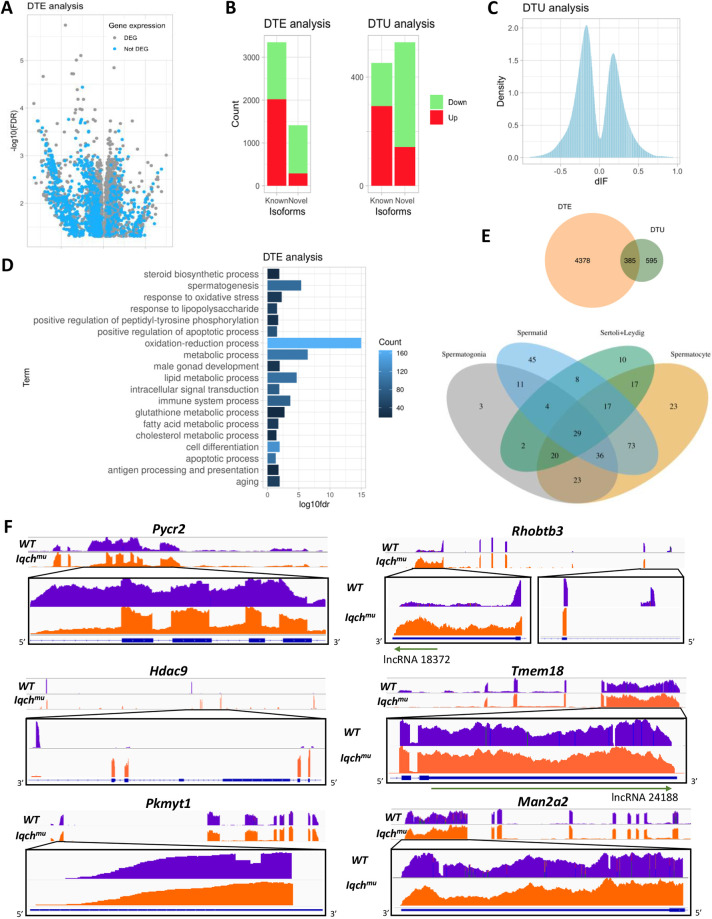
**Differential transcript expression (DTE) and differential transcript usage (DTU) analyses.** (A) Volcano plot of differentially expressed transcripts (DTE analysis), showing which of the genes are also differentially expressed. (B) Bar plot of novel and known isoforms of DTE and DTU, with colours indicating upregulation or downregulation. (C) Density histogram showing the distribution of dIF of switching isoforms. (D) The top 10 enriched GO-BP terms of differentially expressed transcripts (DTE analysis). (E) Isoform fraction ratios of transcripts showing isoform switching and a dIF>60% (DTU analysis). (F) Sashimi plots indicate some differentially expressed isoforms. For each gene, a general view of the coverage calculated for wild type (violet) and *Iqch^mu^* (orange) is depicted in the top panel. The bottom panel shows a zoomed region of the alternative splicing event. Read counts are shown using an identical scale in all samples. The canonical isoform is shown in blue below the panel. LncRNAs in the *Rhobtb3* and *Tmem18* genes are indicated by green arrows.

Our DTE analysis identified 4763 isoforms belonging to 3575 genes showing differential expression. Many of these genes (2652) were found to be differentially expressed in the previous analysis (Fig. 6A). Differentially expressed transcripts seemed to group in the volcano plot, such that those showing greater differences in expression or fold changes were separated from those showing less difference. There was no difference in the number of downregulated isoforms (2458) and upregulated isoforms (2305) (Table S7), and a high proportion of these upregulated DETs were also DEGs (Fig. 6A). Of the DETs identified, 1414 were novel (Fig. 6B), and among these, 533 remain unassigned to a gene. Genes showing the altered expression of five or more isoforms were *Apoe*, *Bscl2*, *Gm2762*, *Gpr19*, *Nt5c1b*, *Odf2*, *Qrich2*, *Tmem144*, *Tmem176b* and *Trim11*, as well as *Iqch* (all isoforms containing exon 19 lost their exons upstream of the cut introduced by CRISPR-Cas9 in that exon)*.* Genes showing DTE were functionally characterized, indicating their role in metabolic, and in cell differentiation and development processes (Fig. 6D). The top significant GO biological process terms were ‘spermatogenesis’, ‘oxidative stress response’ and ‘oxidation-reduction’.

In the DTU analysis, 620 genes featured isoform switch and, specifically, 980 isoforms had an adjusted *P*<0.05 and a difference in the isoform fraction (dIF) greater than 10% (Fig. 6B and Table S7). Isoform fraction values quantify the proportion of parent gene expression originating from a specific isoform, and were calculated as isoform_expression /gene_expression (Fig. 6C). There were 528 novel transcripts, and 452 previously annotated transcripts with DTU, so the presence of unannotated transcripts was very significant (Fig. 6B). In both DTE and DTU analyses, the novel isoforms identified were mostly downregulated in the mutant testis, whereas the known isoforms identified were mainly upregulated, indicating the presence of wild-type *Iqch*-produced novel isoforms; in the presence of *Iqch^mu^*, there was a preferential expression of known isoforms (Fig. 6B and Table S7).

Of the 620 genes identified by DTU showing isoform switch, 308 experienced a significant change in the isoform ratio of one isoform, 246 genes underwent a switch between two isoforms and 45 genes underwent a switch among three or more isoforms. Notably, several transcriptional regulators (including *Ascc2*, *Csrnp2*, *Gon4l*, *Hdac9*, *Rps3*, *Zfp61* and *Zfp820*), factors with a role in spermatogenesis (*Gata4*, *Hfm1*, *Tbc1d20*, *Acox1*, *Adad1*, *Alkbh5*, *Cep131*, *Cfap54*, *Diaph3*, *Prdx4*, *Setx*, *Spata16*, *Spata5*, *Spata9*, *Tesk1*, *Txndc2*, *Tsnax*, *Tdrd5* and *Tdrd7*), and post-transcriptional regulator splicing factors (including *Syf2*, *Tardbp*, *Wtap*, *Alkbh5*, *Cpsf6*, *Mbnl2*, *Pabpn1*, *Snrpf*, *Sympk*, *Tra2a*, *Tfip11* and *Zmat5*) expressed a specific isoform of testis *Iqch^mu^* that was different from the isoform expressed in wild-type, indicating that a subset of AS changes likely resulted from alterations in these factors and their respective downstream targets (Table S7). Some of the genes showing isoform switch were involved in phosphorylation, Golgi organization, transcription, cell differentiation, spermatogenesis and the cell cycle. Among the isoforms showing the larger differences, we found relevant genes for spermatogenesis and fertility, such as *Meiob* and *Ccdc34*. In addition, many of these genes were testis specific, including *BB014433*, *Fam71b* (*Garin3*), *Gm4181*, *Tcp10b* and *Nipsnap3a*. Overall, the integration of both analyses resulted in the identification of 385 isoforms of 336 genes with DTU and DTE (Fig. 6E). These genes, showing both differential isoform expressions and ratios, were expressed mainly in spermatocytes and spermatids (Fig. 6E), suggesting that differential splicing gives rise to different proteins in *Iqch^mu^* throughout meiosis and spermiogenesis that may lead to defective spermatozoa.

**Fig. 7.**
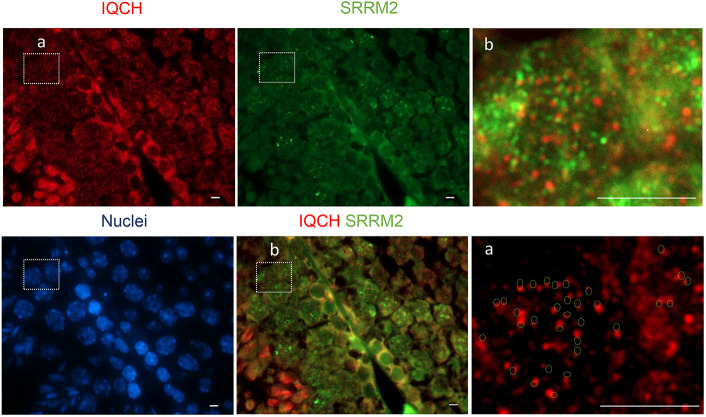
**Nuclear IQCH juxtaposes with the spliceosome marker SC35 (SRRM2).** Representative sections of adult mouse testicles immunostained for IQCH (red) and SC35 (green). In the enlargements of the outlined areas a and b, IQCH spots can be seen juxtaposed with SC35 dots (b); in a, this is represented by the circles indicating the localization of SC35 from b. Scale bars: 10 μm.

Fig. 6F provides six examples of DTU in wild-type versus *Iqch^mu^* mice. We detected IR of three introns of pyrroline-5-carboxylate reductase family member 2 (*Pycr2*) in wild-type testis that were not observed in *Iqch^mu^* testis, which instead expressed the canonical variant. Additionally, the differential expression of histone deacetylase 9 (*Hdac9*) isoforms observed involved the absence of four annotated exons and the presence of a new one in the wild-type testis that did not appear in the canonical form expressed in the mutant testis. We also found a new intron of 20 nucleotides of protein kinase membrane-associated tyrosine/threonine 1 (*Pkmyt1*) in the first exon in wild-type testis that was not present in the canonical isoform of *Iqch* mutants. Another gene with an isoform switch is Rho-related BTB domain containing 3 (*Rhobtb3*), which localizes at the Golgi apparatus and endosomes, and is involved in vesicle trafficking and targeting proteins for degradation in the proteasome (Lutz et al., 2014), for which we observed a new exon between exons 7 and 8 in wild-type mice that were not present in the canonical isoform expressed in *Iqch^mu^* testis. Furthermore, the 3′ untranslated region (3′ UTR) was expressed at higher levels in *Iqch^mu^* compared with wild-type testis. Moreover, in this region of *Rhobtb3* there is a lncRNA of 656 nucleotides (*Mus musculus* lncRNA NONMMUT018372.2) that was expressed at higher levels in the *Iqch^mu^* mutant testis. The other two genes shown in Fig. 7F, transmembrane protein 18 (*Tmem18*) and mannosidase alpha class 2A member 2 (*Man2a2*), featured the same modification in the isoform expressed in the wild type: the appearance of introns in the 3′UTR region.

### IQCH colocalizes with SRRM2 and ERSP1

**Fig. 8.**
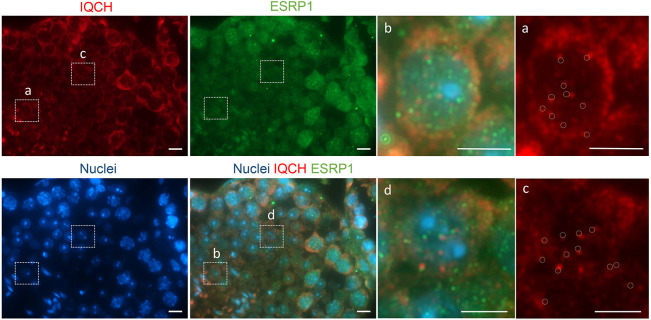
**Nuclear IQCH juxtaposes with ESRP1.** Representative sections of adult mouse testicles immunostained for IQCH (red) and ESRP1 (green). The outlined areas are shown on the right (a-d). IQCH spots (a,c) juxtapose with ESRP1 dots (b,d). In a and c, this is represented by the circles indicating the localization of ESRP1 from b and d, respectively. Scale bars: 10 μm.

**Fig. 9.**
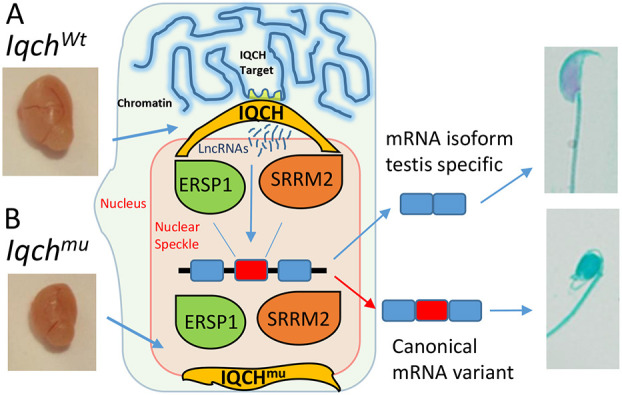
**Model summarizing the effects of *Iqch^mu^* on the regulation of testis-specific transcript isoform expression in mice.** During spermatogenesis, specific mRNA isoforms are expressed in the testis. (A) IQCH localized close to both SRRM2 and ESRP1 (proteins that regulate AS during spermatogenesis associated with co-transcriptional and post-transcription splicing). (B) A deletion produced by Crispr in *Iqch* produces an alteration in the isoforms expressed in the testis, losing the expression of the testis-specific isoform and expressing the principal canonical isoform that is expressed in other tissues.

The effect of *Iqch^mu^* on AS and the nuclear speckled pattern of reactivity seen in spermatocytes and spermatids suggested that IQCH could be related to the spliceosome. To investigate this further, we examined whether IQCH colocalized either with SRRM2 (serine/arginine repetitive matrix 2), a large (∼300 KDa) spliceosome-associated protein ubiquitously expressed that localizes to nuclear speckles, and that promotes interactions between mRNA and the spliceosome catalytic machinery (Blencowe et al., 1994), or with ERSP1 (epithelial splicing regulatory protein 1), which also regulates AS during spermatogenesis and has been associated with co-transcriptional splicing (Saeidi et al., 2018). We found that IQCH localizes in the nucleus attached to both SRRM2 and ESRP1 (Figs 7 and 8). At higher magnification (Figs 7 and 8, images on the right), we can observe that these IQCH speckles were juxtaposed with SRRM2 and ERSP1, suggesting an interaction between these structures. In order to confirm the region of the nucleus where SRRM2 and ESRP1 are located, and because both antibodies were made in mice and therefore could not be used at the same time, we performed colocalization of a U2AF1 [placed within the nuclear speckles, sites of spliceosome assembly (Chusainow et al., 2005)] with SRRM2 and ESRP1 in wild-type and *Iqch^mu^* testis. We can see in wild type that both proteins colocalize with the spliceosome marker U2AF1 within nuclear speckles (Fig. S14). However, when we repeat the immunofluorescence analysis in *Iqch^mu^* testis, we found similar results of colocalization between U2AF1 and SRRM2, but ESRP1 did not localize with U2AF1 (Fig. S15). Based on these results, we propose that IQCH could be a component of nuclear body structures, where it could be associated with co-transcriptional splicing of pre-mRNA to produce the specific required isoforms of spermatogenesis immediately before they ingress in the spliceosome to carry out post-transcriptional splicing (Fig. 9).

Using the Pathway Commons Protein-Protein Interactions Dataset from Harmonizone (https://maayanlab.cloud/Harmonizome/gene_set/IQCH/Pathway+Commons+Protein-Protein+Interactions), we identified 54 proteins (Table S8) showing physical interaction with human IQCH. In addition, using the DAVID Functional Annotation Clustering Tool for these 54 genes, we found clusters of (1) RNA-binding, poly(A) RNA binding and ribonucleoprotein complex; (2) RNA recognition motif domain and nucleic acid binding; (3) mRNA processing, spliceosome and mRNA splicing; (4) double-stranded RNA-binding; and (5) mRNA 3′-UTR binding (Fig. S16).

## DISCUSSION

In this study, we found that IQCH mutant protein prevents normal sperm production in the mouse, largely because of a developmental block during spermiogenesis caused by the dysregulated expression of genes and mRNA isoforms. Alternative splicing of RNA enables the production of multiple gene products from a single gene, thus increasing transcriptome and proteome diversity, which are essential for cell and tissue differentiation and the alterations of which give rise to many diseases. However, the key factors that control tissue-specific alternative splicing remain largely undefined. Testicular cells show extremely high levels of transcription and transcriptome complexity, and the number of AS events and the complexity of mRNA isoforms in spermatogenic cell types exceeds that of most whole tissues (de la Grange et al., 2010; Soumillon et al., 2013). This complexity is thought to result from epigenetic changes that favour specific gene expression during spermatogenesis to generate sperm. However, issues such as why AS is crucial in these cell types, how AS is developmentally controlled and the role of some specific genes regulating these events are largely unknown (Licatalosi, 2016). Here, we show that *Iqch*, a testis-specific gene conserved in all mammals, birds, fish and frogs examined to date, is a strong candidate as a regulator of the expression of specific isoforms during spermatogenesis. *Iqch^mu^* also produces a reduction in the spermatid population that could be contributing to the transcriptomic differences identify, leading to subtle differences in some spermatid genes. However, the DTU analysis, which tests for proportional differences in the expressed isoform composition of a gene, overcomes this issue. Therefore, our results indicate true isoform-switching events as a result of impaired RNA processing.

Pre-mRNA spicing is mediated by a large ribonucleoprotein complex known as the spliceosome. Numerous proteins interact with the spliceosome to regulate splicing and tissue-specific AS. During spermatogenesis, many nuclear RNA-binding proteins (RBPs) and spliceosome-associated proteins are expressed to control AS [RBMXL2 (Ehrmann et al., 2019), PRBP2 (Hannigan et al., 2017), RBM5 (O'Bryan et al., 2013), SAM68 (Paronetto et al., 2011), T-STAR (Ingrid and Elliott, 2010) and CELF1 (Kress et al., 2007)], but the extent to which *Iqch* functions in cooperation with RBPs or as an RBP antagonist remains to be determined. In this regard, the role of some splicing factors in spermatogenesis can be related to the changes observed in *Iqch^mu^* mutant testis. Through a loss-of-function approach, most of them were found to block spermatogenesis either at the spermatocyte or at the spermatid stage, completely or partially. For example, the seminiferous tubules of *Zrsr1* mutant mice had fewer spermatids due to increased intron retention of both U2- and U12-type introns (Horiuchi et al., 2018). Mice lacking *Rbm5* exhibit a similar phenotype, with a spermatid differentiation arrest (O'Bryan et al., 2013), as well as ablation of *Ptpb2* (Hannigan et al., 2017). Loss of *Rbmxl2* disrupts meiosis due to splicing defects, leading to the depletion of spermatids. Knockout of *Sam68* also impairs meiotic progression. Many of these seem to be affecting the mitotic-to-meiotic transition: a period when the splicing is reprogrammed (Hannigan et al., 2017). Along the transition from spermatocyte to spermatid, AS isoforms are mostly constant. All this, together with our results, suggests that aberrant splicing in *Iqch^mu^* could start at the spermatocyte stage, causing accumulation of abnormal isoforms throughout spermiogenesis and finally leading to defective spermatids.

We found that IQCH appears in juxtaposition with the nuclear speckle marker SRRM2 of the spliceosome and in juxtaposition with the splicing regulator ESRP1, suggesting some level of interaction. SRRM2 and ESRP1 colocalize with the spliceosome U2AF1 in wild-type testis, whereas in *Iqch^mu^* testis, ESRP1 fails to colocalize with U2AF1, suggesting that the IQCH regulation of the production of testis-specific isoforms may be mediated by ESRP1. However, although IQCH was not found to be a spliceosome component, the presence of *Iqch* determines the expression of testis-specific isoforms at an earlier stage. ESRP1 regulates AS in epithelial tissues, it is an essential splicing factor required for the regulation of human pluripotency and differentiation, and is highly expressed in spermatogonia, and to a lesser extent in pachytene spermatocytes and round spermatids (Saeidi et al., 2018). It has been reported that SRRM2 regulates spermatid development, acrosome biogenesis and spliceosome activities in bull testis (Selvaraju et al., 2021). In addition, it has been described that SRRM2 acts as a scaffold to organize nuclear speckles, regulating alternative splicing in innate immunity and cell homeostasis (Xu et al., 2022). Interestingly, in these myeloid cells, ESRP1 was downregulated upon SRRM2 knockdown, but no other splicing factors were affected, suggesting that ESRP1 downregulated could account for some of the splicing changes described, principally skipping of cassette exons with short introns (Xu et al., 2022).

In agreement with previous studies, we identified exon skipping as the most common AS event (Schmid et al., 2013) and observed that mRNA isoform switches occur mainly between meiotic spermatocytes and post-meiotic spermatids (Naro et al., 2017). *Iqch^mu^* led to high RI in the testis; however, *Iqch^mu^* also disrupted the parallel expression of many downstream target genes, both at the splicing and transcriptional levels. This makes it difficult to determine whether the phenotype detected in *Iqch^mu^* mouse testis is caused by the aberrant expression of a single target RNA or is a compound phenotype involving multiple genes. What is truly evident is the effect of *Iqch^mu^* on lncRNAs. We observed that lncRNAs are particularly downregulated in spermatids of *Iqch* mutants (Fig. 4C, Table S3A). The vast majority of testicular lncRNA genes are expressed at postmeiotic stages (i.e. spermiogenesis), and are characterized by extensive post-transcriptional regulation, round spermatids being the cells containing the highest numbers of lncRNAs among the different testicular cell populations (Trovero et al., 2020). Owing to transcriptional silencing at later spermiogenesis stages, spermatids have developed a panoply of mechanisms of post-transcriptional regulation as a strategy to later regulate the synthesis of proteins subsequently required by elongated spermatids and sperm (Geisinger, 2008; Gan et al., 2013). Some regulatory mechanisms could be binding of repressors to UTRs of testis-specific transcripts, mRNA sequestration as free ribonucleoprotein particles, regulation of poly(A) tail lengths, regulation of AS by interacting with specific splicing factors, forming RNA-RNA or RNA-DNA duplexes, or affecting chromatin remodelling (Kleene, 2001; Romero-Barrios et al., 2018; Pisignano and Ladomery, 2021). It has been reported that lncRNAs directly or indirectly regulate the AS events of downstream target genes, thus affecting the occurrence of cancer, development, tissue differentiation, nuclear organization, etc. (Ouyang et al., 2022; Pisignano and Ladomery, 2021). We suggest that the control of the expression of many isoforms during spermatogenesis by the mutant *Iqch* could be mediated by downregulating the expression of lncRNAs (Fig. 9).

Our analyses suggest a model whereby *Iqch* controls a network of AS events in postmitotic germ cells (spermatocytes and spermatids) that is necessary to establish the correct protein isoforms required for spermatid development. We propose that spermatogenic failure in *Iqch* mutant testis is the consequence of an aberrant accumulation of AS isoforms across the large network of functionally interconnected lncRNAs and proteins. This process subsequently alters the expression of mRNAs, lncRNAs and specific isoforms of spermatogenesis. In summary, we show that isoform expression is highly and specifically regulated in the testis, presenting evidence that this regulation is essential for spermatogenesis, and define a network of specific isoforms with roles in Golgi organization, spermatogenesis, splicing, transcription regulation and poly(A) RNA binding, which are coordinately regulated by *Iqch*-dependent AS. Our findings significantly increase the number of known genes and specific isoforms of spermatogenesis and offer important insight into the physiological functions of an AS program in the mammalian germline, as well as direction for unravelling connections among *Iqch*-dependent AS, the male germline and fertility.

## MATERIALS AND METHODS

### Generating *Iqch* mutant mice

To target the mouse *Iqch* gene, Cas9 mRNA and sgRNAs (Table S9) were produced using the mMESSAGE mMACHINE T7 Ultra Kit and GeneArt Precision gRNA Synthesis Kit (Thermo Fisher Scientific), respectively. The resultant RNAs were then injected into B6CBAF1 (C57BL/6xCBA) zygotes, which were transferred to pseudo-pregnant females (Gómez-Redondo et al., 2020). Pups were genotyped by PCR conducted in standard conditions with *Iqch* primers (Table S9) and screened for mutations using the SURVEYOR Mutation Detection Kit (Transgenomic). Founders were confirmed by Sanger sequencing. Two edited lines were successfully generated with deletions that were not multiples of three nucleotides (which alter the amino acid sequence of the protein). Homozygotes were used in all experiments. Wild-type mice were used as controls. Animal experiments were conducted following European legislation. All study protocols were approved by the Ethics Committee on Animal Experimentation of the INIA (Madrid, Spain) (September 21, 2015) and were registered at the Direccion General de Agricultura y Ganadería de la Comunidad de Madrid (Spain) (PROEX 137.2/21). Throughout the manuscript we use ‘*Iqch^mu^*’, to refer to results of the two selected lines, which always carried the mutation in homozygosity.

### Sperm collection

Wild-type and *Iqch^mu^* males were euthanized by cervical dislocation. Spermatozoa were collected by squeezing the vasa deferentia and dissecting the caudal epididymides. Spermatozoa were allowed to swim-out for 30 min at 37°C in a droplet of 500 μl HTF medium [2.04 mM CaCl_2_, 101.6 mM NaCl, 4.69 mM KCl, 0.37 mM KH_2_PO_4_, 0.2 mM MgSO_4_, 21.4 mM sodium lactate, 0.33 mM sodium pyruvate, 2.78 mM glucose, 25 mM NaHCO_3_, 100 U/ml penicillin, 50 μg/ml streptomycin and 0.001% (w/v) phenol red, supplemented with 1% (w/v) BSA]. The concentration of the sperm sample was determined in a Thoma cell counting chamber. Sperm viability was assessed through eosin/nigrosine staining, examining 200 cells.

### Sperm morphology

To assess sperm morphology, 6 μl of sperm suspension was spread on a slide and stained using the Spemac Kit (minitube) following the manufacturer's indications. The head of the sperm cell appears red, the acrosome, centrepiece and tail green, and the equatorial zone is pale green, allowing for the identification of different abnormalities. These were classified into the categories: normal morphology, neck and midpiece defects, head defects and tail defects. The slides were observed under a light microscope (Nikon Eclipse E400), analysing at least 200 cells per male from wild-type animals and all the sperm cells from *Iqch^mu^* males.

### Sperm motility

Six microlitres of sperm suspension were placed in a Mackler chamber on the stage of a microscope heated to 37°C (Nikon Eclipse E400) and fitted with a digital camera (Basler acA1300-200uc). Five videos of 1.5 s each were recorded and analysed using the Integrated Semen Analysis System (ISAS 2008). The parameters analysed were as described by Perez-Cerezales et al. (Pérez-Cerezales et al., 2018): straight-line velocity (VSL) (time-averaged velocity of the sperm head along a straight line from its first position to its last position, expressed in μm/s); curvilinear velocity (VCL) (time-averaged velocity of the sperm head along its actual curvilinear path, expressed in μm/s); average path velocity (VAP) (velocity over an average path generated by a roaming average between frames, expressed in μm/s); linearity (LIN) (defined as (VSL/VCL)×100); straightness (STR) [defined as (VSL/VAP)×100]; wobble (WOB) [defined as (VAP/VCL)×100]; the amplitude of lateral head displacement (ALH) (width of the lateral movement of the sperm head, expressed in μm) and beatcross frequency (BCF) (number of times the sperm head crosses the direction of movement per second, expressed in Hz).

### Gonadosomatic index

The testes were extracted from each individual and weighed to establish the relationship between gonad and body size, a measure known as the gonadosomatic index (GSI).

### Western blots

Samples from the testes of wild-type and *Iqch^mu^* mice were lysed in 300 μl of RIPA buffer containing 50 mM TrisHCl (pH 7.6), 150 mM NaCl, 1% Triton X-100, 0.5% sodium deoxycholate and 0.1% SDS supplemented with cOmplete, EDTA-free Protease Inhibitor Cocktail (Roche) for 1 h at 4°C. The lysate was centrifuged at 12,000 **g** for 15 min, and the supernatant was collected for protein analysis. Total protein was quantified using the Pierce BCA Protein Assay Kit (Thermo Fisher Scientific) following the manufacturer's instructions. Proteins were run on an SDS-PAGE gel (8% acrylamide loading 50 µg of total protein per well) and transferred to a nitrocellulose membrane for immunoblotting following standard procedures. Blocking was conducted with 3% BSA in PBS-T (0.05% Tween) and membranes were incubated overnight at 4°C with a rabbit polyclonal anti-Iqch antibody (04030000809; ChinaPeptides; again sequence KAIKRIKNLIRGEEAYIVGG from 596 to 615 amino acids of IQCH) diluted 1:500. Incubation with the secondary antibody, goat anti-rabbit IgG-HRP (Santa Cruz Biotechnology, sc-2004) diluted 1:5000, was performed for 2 h at room temperature. As a control, we used the monoclonal anti-β-actin peroxidase-conjugated mouse antibody (A3854, Merck) diluted 1:5000. The chemiluminescence signal was digitalized using an ImageQuant LAS 500 chemiluminescence CCD camera (GE Healthcare Life Sciences, 29005063).

### Protein structure prediction of IQCH

Murine IQCH protein disordered region and IQ domain positions are described according to Uniprot ID Q9D2K4-1, for the wild-type and mutants 5 and 7. DNA- and RNA-binding domains have been identified by the PredictProtein online server (Bernhofer et al., 2021), after introducing the sequence of the protein of interest. Tridimensional protein structure prediction was generated with the AlphaFold AI system (Varadi et al., 2022) developed by DeepMind, and visualized by Ezmol online viewer (Reynolds et al., 2018).

### Immunohistochemical study

Routine histology was carried out on experimental and control testes. Briefly, testes were fixed in 4% (w/v) paraformaldehyde and embedded in paraffin wax. Sections (4 μm) from wild-type and *Iqch^mu^* males were de-waxed, rehydrated and stained with Hematoxylin and Eosin.

To detect IQCH, sections were firstly de-waxed and rehydrated. To retrieve the antigen, the slices were incubated in a buffer containing 10 mM sodium citrate and 0.05% at 100°C for 20 min. After washing with distilled water, the sections were permeabilized in 1% PBS-T (Triton X-100) for 20 min. Slides were then blocked in PBS supplemented with 3% BSA and 0.1% Tween for 1 h in a humid box. Next, they were incubated overnight at 4°C with anti-Iqch antibody (STJ194004; Saint John's Laboratory) diluted 1:200 in blocking solution. Following three washing steps with PBS, the slides were incubated with a goat anti-rabbit secondary antibody Alexa Fluor 568 (Invitrogen) at 37°C for 1 h followed by washing three times with PBS, and staining of the acrosome and nuclei with 15 μg/ml FITC-PNA and 6.5 μg/ml Hoechst, respectively. VECTASHIELD Mounting Media for Fluorescence (Vector Laboratories) was used for observation using a Nikon Eclipse TE-2000 confocal microscope. To assess the colocalization of IQCH with SRRM2 or ESRP1, the same procedure was performed incubating the sections with a combination of antibodies: anti-IQCH (STJ194004; Saint John's Laboratory; 1:250), anti-SC-35 (S4045, Sigma-Aldrich; 1:500) and anti-ESRP1 (27H12.F3.H5, Thermo Fisher Scientific; 1:250). The speckle-marking antibody S4045, which was originally raised against spliceosomal extract (Fu and Maniatis, 1990), colocalizes with other speckle markers (Ilık et al., 2020) and has recently been determined to predominantly detect the speckle-resident protein SRRM2 between residues 1360 and 1884 (Ilık et al., 2020), although it has been marketed as an antibody against SC35 (also called SRSF2), another speckle-resident protein. To assess the colocalization of U2AF1 and SRRM2 or ESRP1, we used the same protocol described above using anti-U2AF1 (Ab-86305; Abcam; 1:250) instead of IQCH antibody.

### Squashing and spreading of spermatocytes and immunocytology

Testes from adult Iqch knockout were detunicated and seminiferous tubules processed to obtain squashed (Parra et al., 2002) or spread (Peters et al., 1997) preparations of spermatocytes. Afterwards, slides were rinsed three times for 5 min in PBS and incubated overnight at 4°C for simultaneous double immunolabelling with the corresponding primary antibodies diluted in PBS. To detect SYCP3, we employed a rabbit polyclonal anti-hSYCP3 (Abcam, ab-15092) at 1:100 dilution. Kinetochores were revealed with a purified human anti-centromere autoantibody (ACA serum; Antibodies Incorporated, 435-2RG-7) at a 1:20 dilution. For detecting γ-H2AX, we used a mouse monoclonal antibody (Millipore, 05-636) at 1:1000 dilution. Then, slides were rinsed three times for 5 min in PBS and incubated for 1 h at room temperature with the corresponding secondary antibodies. The following secondary antibodies were employed: Alexa 488-conjugated donkey anti-rabbit IgG (Molecular Probes, A-21206), Alexa 594-conjugated donkey anti-mouse IgG (Molecular Probes, A-21203) and Alexa 594-conjugated goat anti-human IgG (Molecular Probes, A-11014). After three rinsing steps in PBS, slides were counterstained with 10 μg/ml DAPI (4′,6-diamidino-2-phenylindole) for 3 min, rinsed in PBS for 1 min, mounted with Vectashield (Vector Laboratories) and sealed with nail varnish. Observations were performed using an Olympus BX61 microscope equipped with epifluorescence optics. Single images or image stacks across complete cells/nuclei were captured with an Olympus DP71 digital camera controlled by the CellSens Dimension software (Olympus). Images were analysed and processed using the public domain ImageJ software (National Institutes of Health, USA; http://rsb.info.nih.gov/ij) and Adobe Photoshop CS5 software.

### Germ cell isolation

We analysed three wild types and three *Iqch^mu^* animals to determine the proportion of n, 2n and 4n chromosome dotation type cells. To do so, we isolated germ cells from 8 months mice testis by enzymatic digestion. Firstly, we got both testis, washed in PBS and put them on a sterile plate. We decapsulated the tubules, tore the albugineum tunique, and sliced them with scissors. We digested the tubules in a 1 mg/ml collagenase solution for 15 min at 37°C. Mechanical desegregation by pipetting was performed to contribute to cell isolation. Cells and non-digested tissue were centrifugated at 200* **g*** for 5 min and washed in PBS. After a second centrifugation, 0.25% trypsin-EDTA digestion was performed to isolate small cell groups for 3 min at 37°C. We blocked trypsin digestion with a medium containing 10% FBS. We filtered the digested mixture to eliminate undigested tissue fragments. To isolate germ cells, we maintained the pool of cells in a p35 gelatinized plate (1%) overnight with DMEM high glucose 10% FBS medium. Sertoli and Leydig cells adhered to the plate, and germ cells stayed in the supernatant. After somatic and gem cell separation, the supernatant was recovered, centrifuged at 200* **g*** for 5 min, and fixed and permeabilised with 30% ethanol/PBS, at 4°C overnight.

### Cell cycle study by cytometer

To perform the cell cycle study, fixed gem cells were stained for 1 h at 4°C with 6.5 μg/ml Hoestch 33342/PBS solution and were analysed in a Cytek Aurora flow cytometer as previously described (Bastos et al., 2005). Three peaks with increasing Hoechst fluorescence could be detected as representative of haploid (1C), diploid (2C) and tetraploid (4C) cells. We used a volumetric measurement during sample acquisition, using a volume sensor, that allows the calculation of events per µl for any given population and thus determine the absolute number of cells in the analysed sample. The data have been acquired and analysed using the SpectroFlo software (Cytek Biosciences).

### RNA-seq and differential gene expression

Total RNA was extracted from four testes of *Iqch^mu^* and four testes of wild-type 4-month-old males using the TRIzol reagent (Invitrogen) and then treated with DNase (Promega) for 1 h. The purified total RNA was stored in nuclease-free water, and then used for first-strand synthesis. RNA concentration was measured using a Qubit RNA Assay Kit in a Qubit 2.0 fluorometer (Life Technologies). RNA-seq libraries were prepared from the follicle pools described above using the TruSeq RNA kit from Illumina according to the manufacturer's recommendations, and cDNA libraries were used for sequencing with an Illumina HiSeq2500 sequencer, generating an average of 54 million 125* *bp paired-end reads per sample.

The quality of raw sequencing data was assessed using FastQC v0.11.7 (https://www.bioinformatics.babraham.ac.uk/projects/fastqc/), and then adapters were filtered and trimmed with Trimmomatic v0.38 (Bolger et al., 2014). Paired end reads were mapped employing Subread aligner v2.0.0 (Liao et al., 2013) against the GRCm39 reference genome and read quantified using FeatureCounts (Liao et al., 2014). Before downstream analysis, genes were filtered out by considering their expression in any of the experimental groups and keeping genes when their count was at least 75 in two or more samples of a group. Then, after the normalization of samples, two different R packages were used for the differential expression analysis: DESeq2 v1.30.1 (Love et al., 2014) and edgeR v3.32.1 (Robinson et al., 2010; McCarthy et al., 2012). Differentially expressed genes (DEGs) were those with an adjusted *P*-value cutoff of 0.01, which overlapped between both methods, improving the reliability of the results. The functional analysis, which involves the Gene Ontology term over-representation analysis, was performed using the DAVID tool (Huang da et al., 2009). To assess the functional characterization of lncRNAs (a relevant gene type of our set of DEGs), we analysed interactions of lncRNAs with proteins and mRNAs, making use of the RNAInter database, which contains *Mus musculus* lncRNA-associated interactions through experimental validation and/or computational prediction (Lin et al., 2020).

### Differential splicing analysis

Differentially spliced events were analysed by making use of rMATS (Shen and Rajender, 2014), which compares PSI (percent spliced in) values (a measure of the exon inclusion level in a sample) between experimental groups to identify alternative splicing events whose PSI values are significantly different. Events were differentially spliced considering a coverage of a minimum of five reads covering the splice event, an FDR of 0.05 and a ΔPSI (difference between PSI values of the experimental groups) of 0.1.

### *De novo* transcript quantification

To analyse novel transcripts in the differential isoform expression analysis, in addition to annotated transcripts, we undertook transcript quantification using Stringtie (Pertea et al., 2015). Previously, the alignment of reads with HISAT2 (Kim et al., 2015) was required. Next, StringTie assembled the read alignments and estimated transcript abundances, which were used in the differential isoform expression analysis.

### Differential isoform expression analysis

StringTie quantification tables were used in the differential transcript expression (DTE) pipeline, which involved the filtering of transcripts with a coverage of five or less in more than two samples for any of the conditions, TMM normalization, and the differential expression test using edgeR (Robinson et al., 2010; McCarthy et al., 2012) and limma voom (Law et al., 2014). Isoforms with an adjusted *P*<0.05 according to both methods were overlapped to obtain the differentially expressed isoforms. In addition, isoform switches between conditions were identified using isoformSwitchAnalyzeR (Vitting-Seerup et al., 2019), which implements the DEXSeq approach to test for differential transcript usage (DTU). An isoform was considered significant when returning a FDR<0.05 and a dIF (difference in isoform fraction or usage) value above 0.1.

### Classification of genes according to their expression in the different testis cell types

To characterize the set of DEGs, we classified these according to their expression levels in the different testis cell types. We integrated data from diverse studies that profiled gene expression during the process of spermatogenesis, revealing marker genes for distinct stages and testis cell types. The selected studies used different approaches or a combination of these: exploiting the first spermatogenic wave, purifying the major spermatogenic cell types and/or single-cell RNA-seq. In particular, we compiled lists of genes associated with each cell type identified in previous studies (Margolin et al., 2014; Ball et al., 2016; Chen et al., 2018; Green et al., 2018; Guo et al., 2018; Hermann et al., 2018; Ernst et al., 2019; Jung et al., 2019; Trovero et al., 2020; Li et al., 2021). In addition, we integrated data from the databases The Human Protein Atlas v20.1 (https://www.proteinatlas.org/humanproteome/celltype) (Uhlén et al., 2015) and the Mouse Cell Atlas v2.0 (Han et al., 2018).

Besides using markers obtained by others, we processed and analysed the single-cell transcriptomic data emerging from the study of Green et al. (2018) using the same reference annotation as our RNA-seq analysis (GRCm39) for a finer-tuned classification. The raw data, which were produced using Drop-seq technology, were downloaded from GEO database (series GSE112393) and the preprocessing steps were performed using Drop-seq tools v2.4.0 from the McCarroll laboratory (Macosko et al., 2015). These tools provide the pipeline to tag reads with cellular and molecular barcodes, to filter and trim starting and poly A sequences, to align them with STAR, to tag reads with gene names, to correct for common Drop-seq errors and, finally, to obtain a digital expression matrix per sequencing run. Next, the Seurat R-package v4.0.1 (Hao et al., 2021) was used for dimensionality reduction, clustering of cells and identification of marker genes. These marker genes were the ones used to extend our classification. Altogether, DEGs were categorized in spermatogonia, spermatocytes, spermatids, Sertoli cells, Leydig cells, other somatic cells (macrophages, endothelial, innate lymphoid, peritubular myoid cells and fibroblasts) and spermatozoa.

## Supplementary Material

10.1242/develop.201334_sup1 Supplementary information
