# Supplementary material for: An ancient testis-specific IQ motif-containing H gene regulates specific transcript isoform expression during spermatogenesis
Source: Development. 2023 Apr 4;150(7):dev201334. doi: 10.1242/dev.201334 (PMC10112910; doi:10.1242/dev.201334)
Supplement: Supplementary information [file develop-150-201334-s1.pdf]

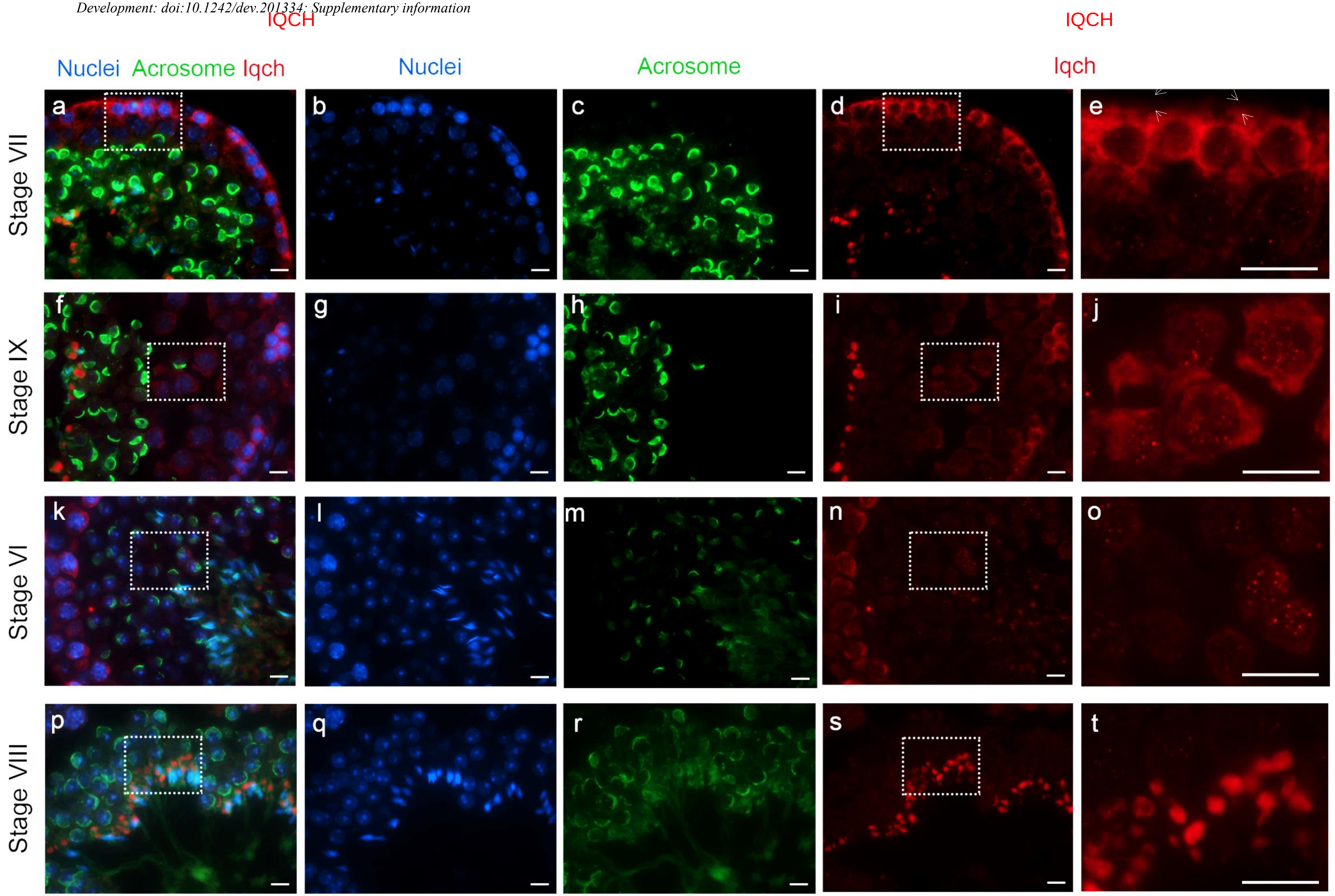

Fig. S1. **Immunolocalization of IQCH during spermatogenesis in mice**. Representative images of cross-sections of seminiferous tubules merged at different stages of spermatogenesis (a, f, k, p), stained with Hoechst (blue: b, g, l, and q), FITC-PNA (green: c, h, m, and r), and anti-IQCH (red: d, i, n, and s). Enlarged pictures of the boxed areas show the distribution of IQCH in the different types of testicular cells: a cytoplasmic location in spermatogonia (e), cytoplasmic and nuclear speckled pattern in spermatocytes (j), a spotted nuclear distribution in round spermatids (o), and a cytoplasmic cumulus in the elongated spermatids (t). The arrowheads indicate low-intensity and few spots visible in the nucleus of primary spermatocytes. Scale bar: 10 μm.

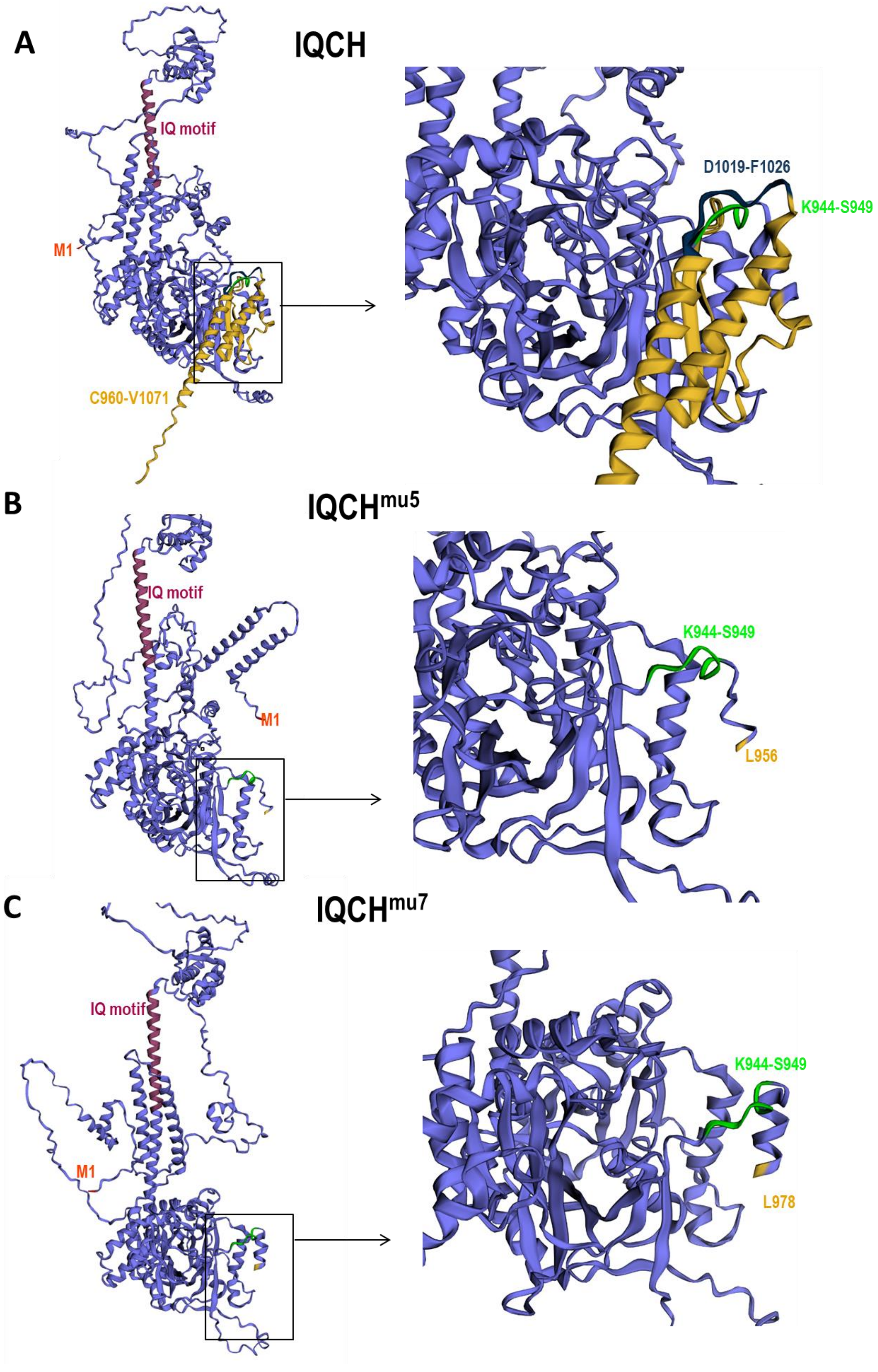

Fig. S2. Protein structure prediction of IQCH wt and mutants using the AlphaFold AI system developed by DeepMind, and visualized by Ezmol. DNA and RNA-binding domains have been described in the Predict Protein webserver. First methionine is featured in red, the IQ motif (K406-A435) in pink, and RNA binding residues (K944-S949) in light green. In A, WT protein corresponds to the Q9D2K4 ID from Uniprot, and it includes also the DNA-binding domain (D1019-F1026) in dark blue in the C-terminal region (C960-V1071, in gold) lost in both IQCH mutants 5 and 7. In B and C, is pointed out in gold the last amino acid, L956 and L978, of mutant 5 and 7, respectively. RNA-binding domain folding, placed in the last residues of the mutant proteins, is modified in both mutants.

Development • Supplementary information

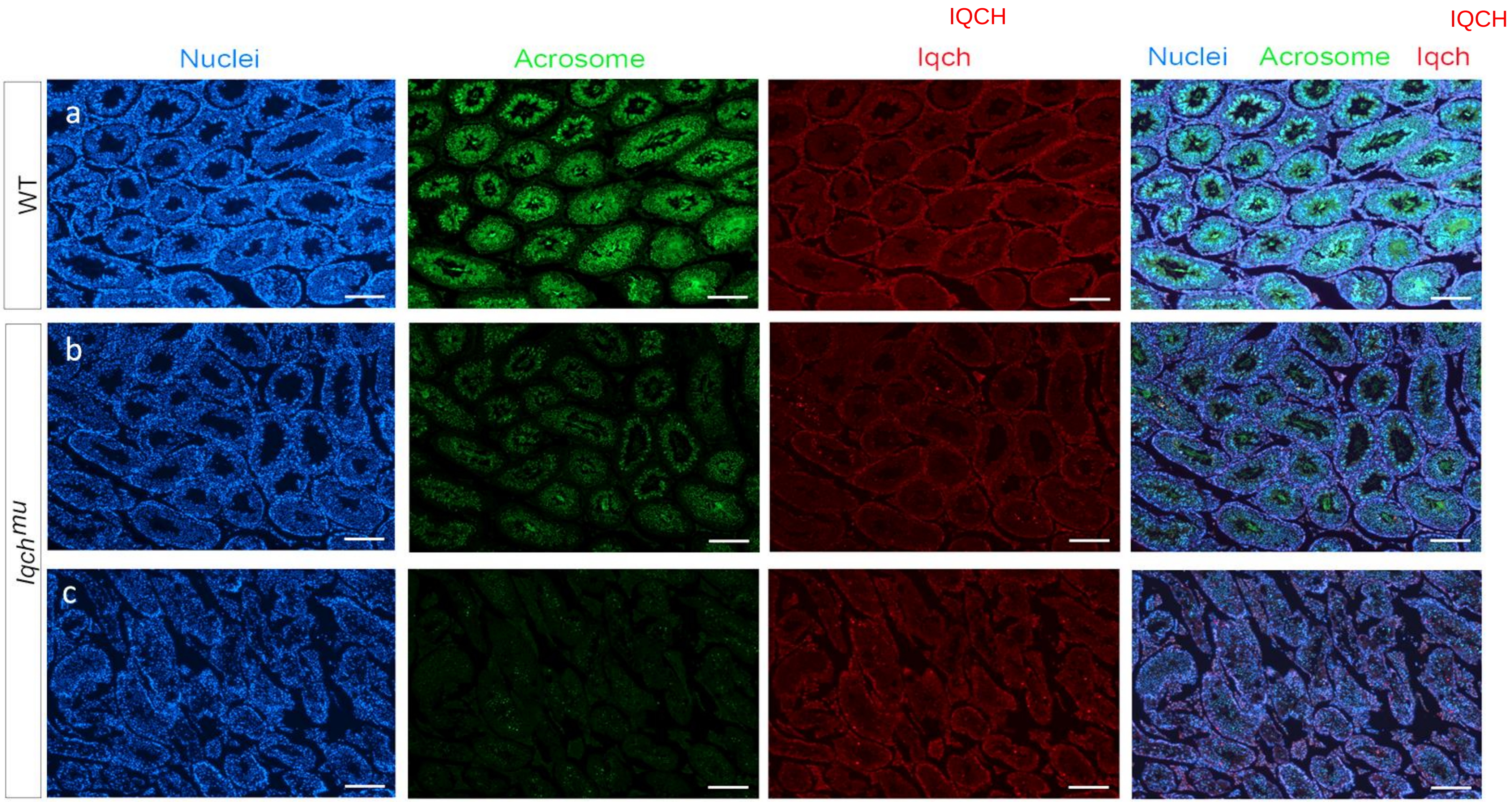

Fig. S3. **Distribution of IQCH in wild-type and *Iqch^{mu}* male mice**. Representative images of cross-sections of seminiferous tubules stained with Hoechst (blue), FITC-PNA (green), and anti-IQCH (red). The images show that *Iqch^{mu}* animals expressed the protein, although the testicular structure of the mutant animal (b, c) is disorganized, containing few acrosome-positive cells. Scale bar: 200 µm.

Development • Supplementary information

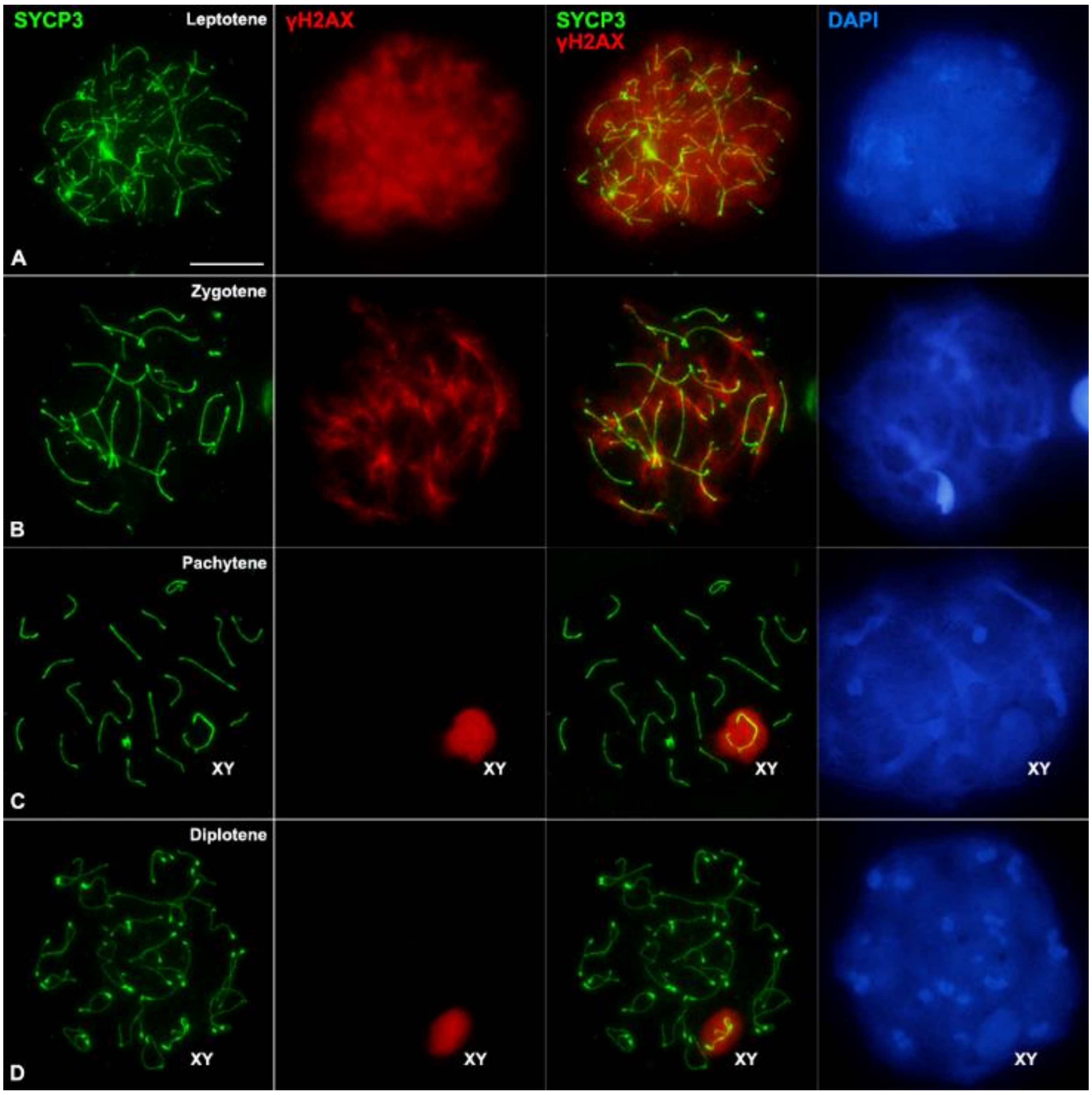

Fig. S4. Synapsis and recombination are not altered in *Iqch^{mu}* spermatocytes. Double immunolabelling of SYCP3 (green) and γH2AX (red) in spread Iqch knockout prophase I spermatocytes. A. Leptotene spermatocyte nuclei presented stretches of SYCP3, as the axial elements of the synaptonemal complex are being formed. γH2AX labelling covered most of the nuclei because of the massive formation of programmed DNA double strand breaks. B. During zygotene, as synapsis progressed, the SYCP3-labelled axial/lateral elements started to pair into thicker filaments. In parallel, γH2AX labelling progressively reduced, due to the progression of meiotic recombination. C. In pachytene spermatocytes autosomal bivalents achieved full synapsis and displayed fully paired SYCP3-labelled lateral elements at their entire length. By contrast, sex chromosomes (XY) only synapsed at one distal end corresponding to the pseudoautosomal region. At this stage γH2AX was mainly found over the chromatin of sex chromosomes (XY). D. During diplotene the SYCP3-labeled lateral elements started to desynapse and γH2AX persisted over the chromatin of sex chromosomes (XY). Bar in A, 10 µm.

Development • Supplementary information

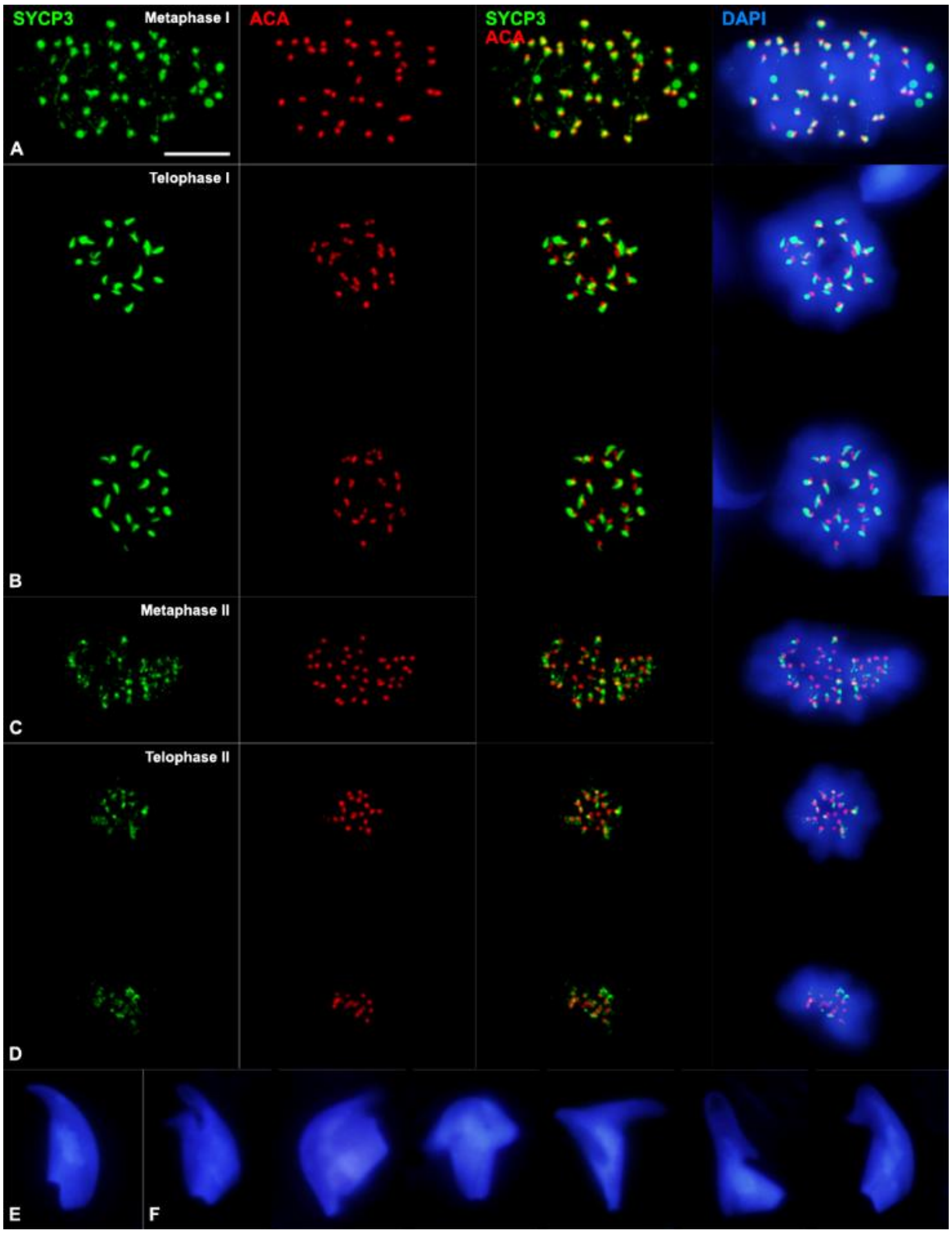

Fig. S5. Chromosome segregation during both meiotic divisions is not altered in *Iqch*^{mu} spermatocytes. Double immunolabelling of SYCP3 (green) and kinetochores (ACA, red) in squashed *Iqch*^{mu} dividing spermatocytes. A. In metaphase I spermatocytes bivalents were correctly aligned at the metaphase plate. SYCP3 was found at the interchromatid domain of bivalents and accumulated at their centromeres, where sister kinetochores appeared closely associated. B. In telophase I homologous chromosomes accurately segregated to opposite cell poles. SYCP3 remnants were found at centromeres, close to sister kinetochores. C. Metaphase II spermatocytes presented chromosomes adequately aligned with SYCP3 remnants at centromeres and sister kinetochores oriented to opposite cell poles. D. In telophase II sister chromatids reached cell poles. E, F. Magnification of selected elongateed spermatids from squashed seminiferous tubules of Iqch knockout mice. Elongated spermatids with regular morphology were formed (E), but some aberrant spermatid morphologies (F) were also present. The images are projections of different focal planes throughout the spermatocyte/spermatid volume. Bar in A, 5 μm.

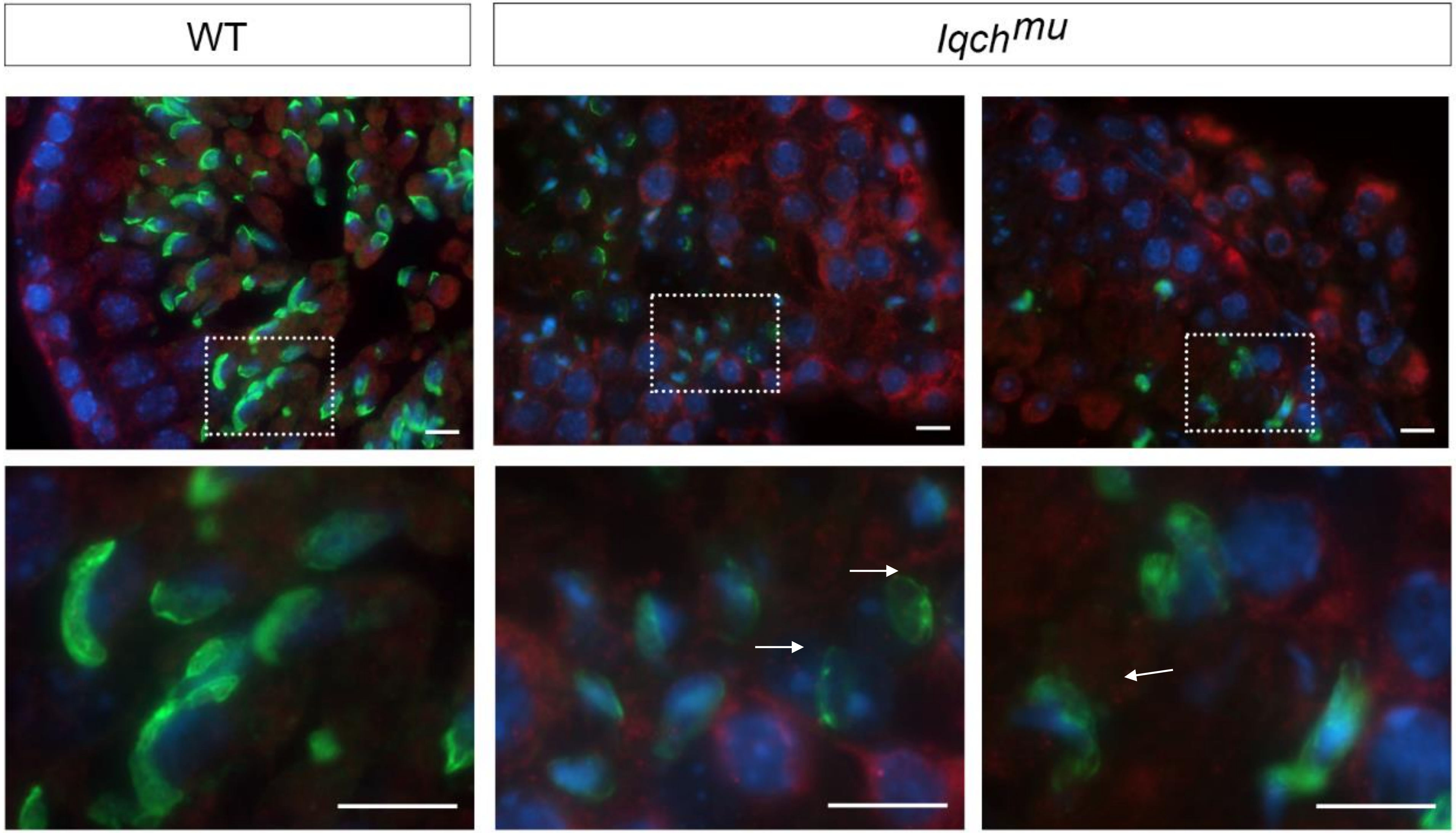

Fig. S6. Abnormal acrosome in *Iqch*^{mu} mouse spermatids. Shown in histochemical staining of FITC-conjugated PNA (green), Hoechst (blue), and anti-IQCH (red), in sections of adult WT and *Iqch*^{mu} mouse testes showing spermatids with acrosome in the acrosome phase. Acrosome are labeled by PNA (green), in WT is showed typical acrosome phase, the acrosome forms the moon-shaped structure covering the nucleus. In *Iqch*^{mu} the acrosome failed to form, resulting discontinuous and fragmented acrosomal structures (arrow). Bottom panels show higher-magnification views of the framed areas in the top panels (scale bar, 10 μm).

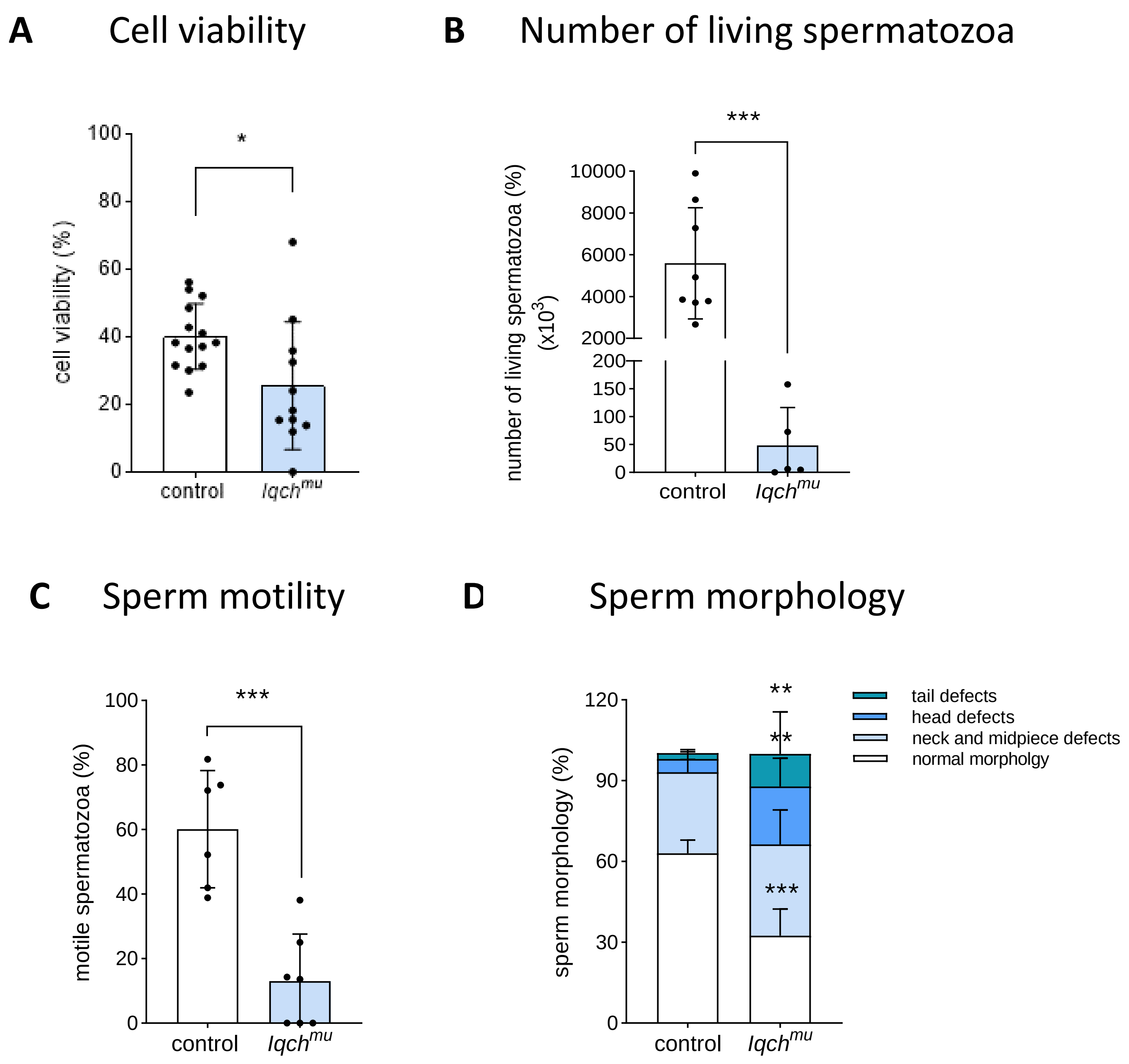

Fig. S7. Sperm characteristics in wild type and *Iqch*[mu] mice. Cauda epididymidis sperm were obtained and analyzed as explain in Materials and Methods. **(a)** Cell viability (n=14 wt, and 10 *Iqch*[mu] mice). **(b)** Number of living spermatozoa (n=8 wt, and 5 *Iqch*[mu] mice). **(c)** Sperm motility (n=6 wt, and 7 *Iqch*[mu] mice). **(d)** Sperm morphology (n=8 wt, and 8 *Iqch*[mu] mice). Data represent the mean ± SEM. * $p<0.01$, ** $p>0.001$, *** $p<0.0001$ (one way ANOVA, Tukey's test).

Development • Supplementary information

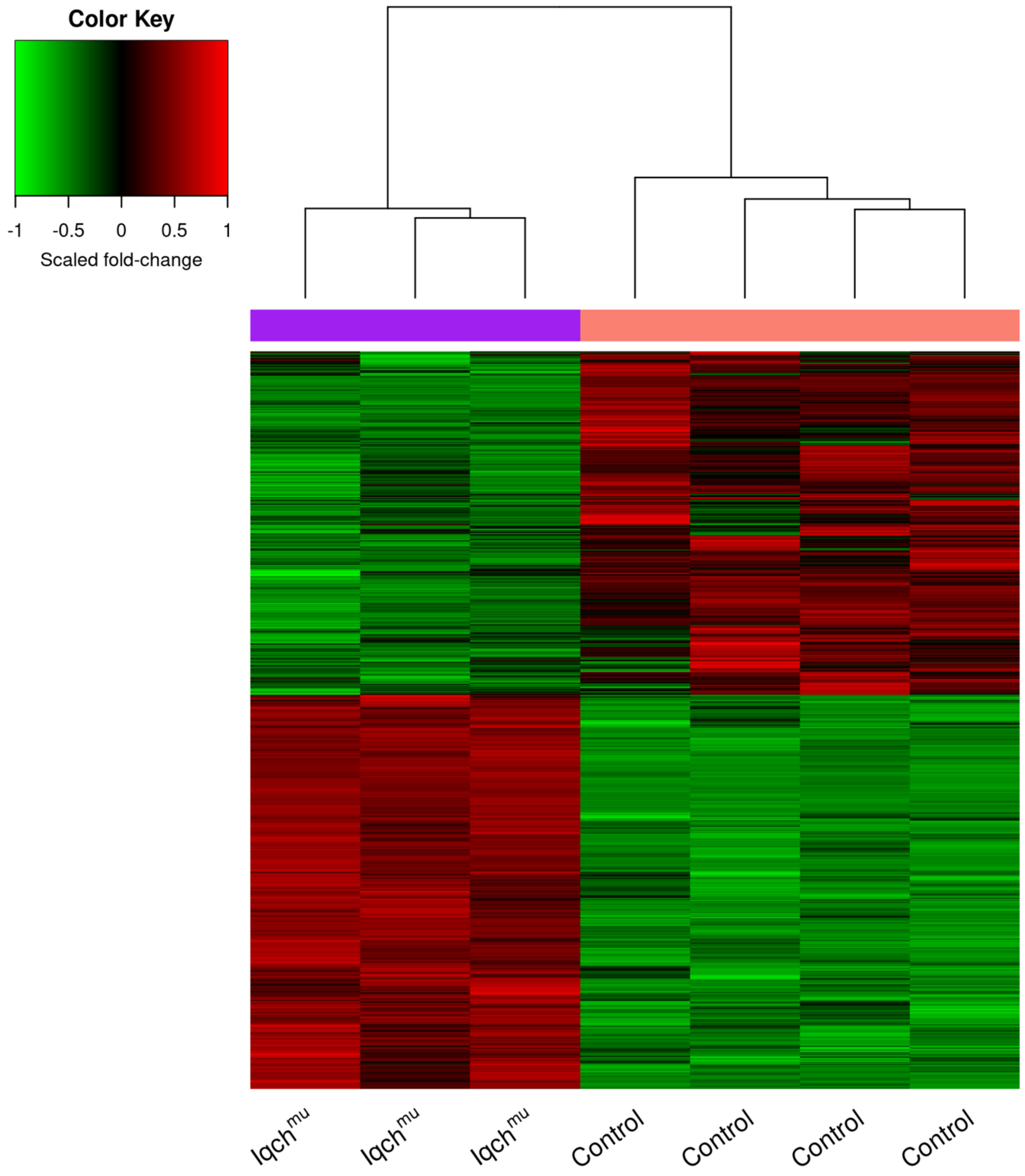

Fig. S8. **RNA-seq analysis.** Hierarchical clustering analysis was performed based on normalized counts of the top DEGs in control and *Iqch*^{mu} mice samples.

Development • Supplementary information

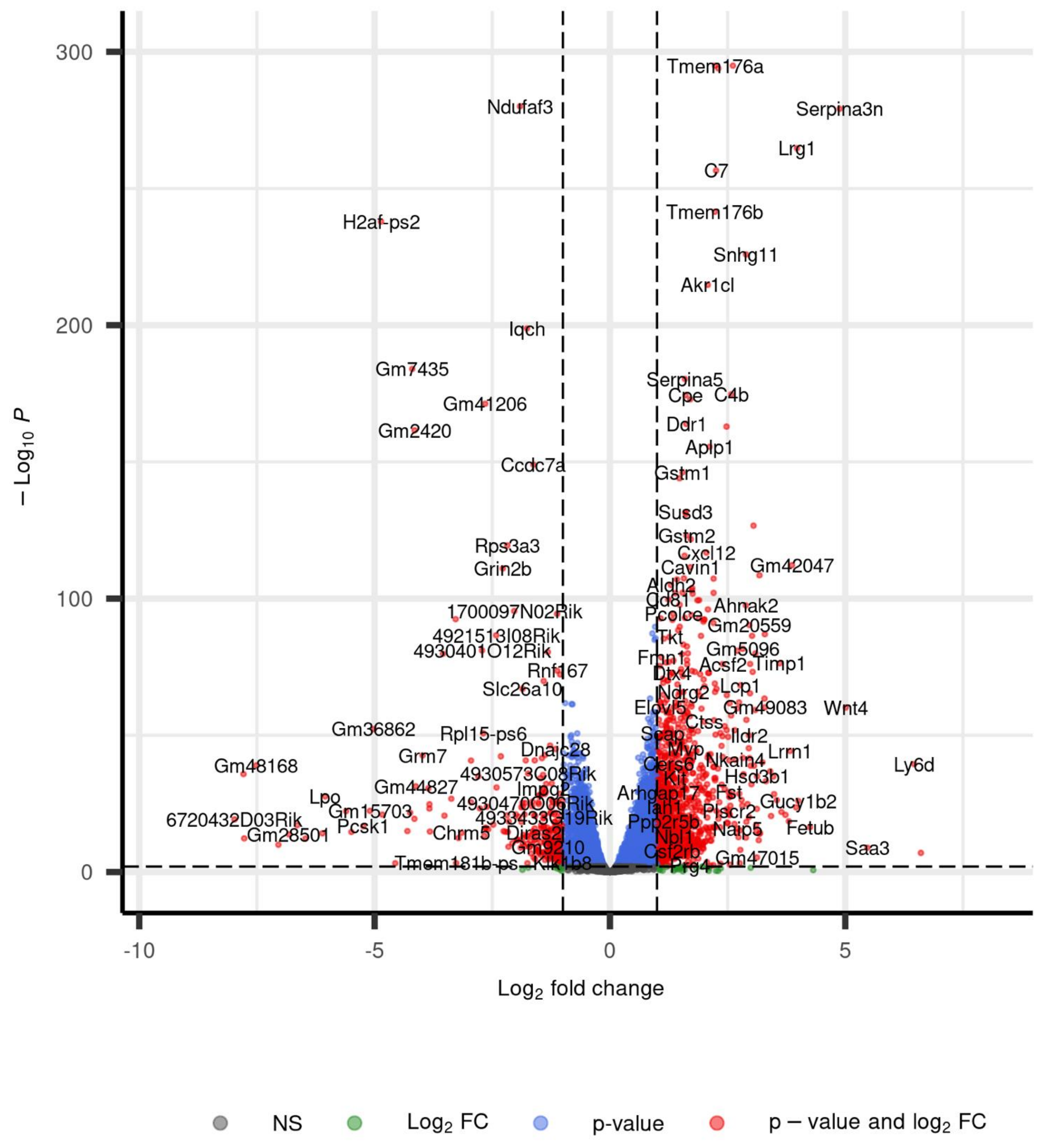

Fig. S9. **RNA-seq analysis.** Differentially expressed genes in testis of control and *Iqch*^{mu} mice are presented as a volcano plot using log values of the fold change and -log10(Padj) values. Each data point represents a single gene, with those in red and blue dots representing DEGs and non-DEGs respectively for a cut off thresholds of |FC| > 2 and Padj value < 0.01; and the blue and black dots represent DEG and non-DEGs respectively for a Padj value < 0.01.

Development • Supplementary information

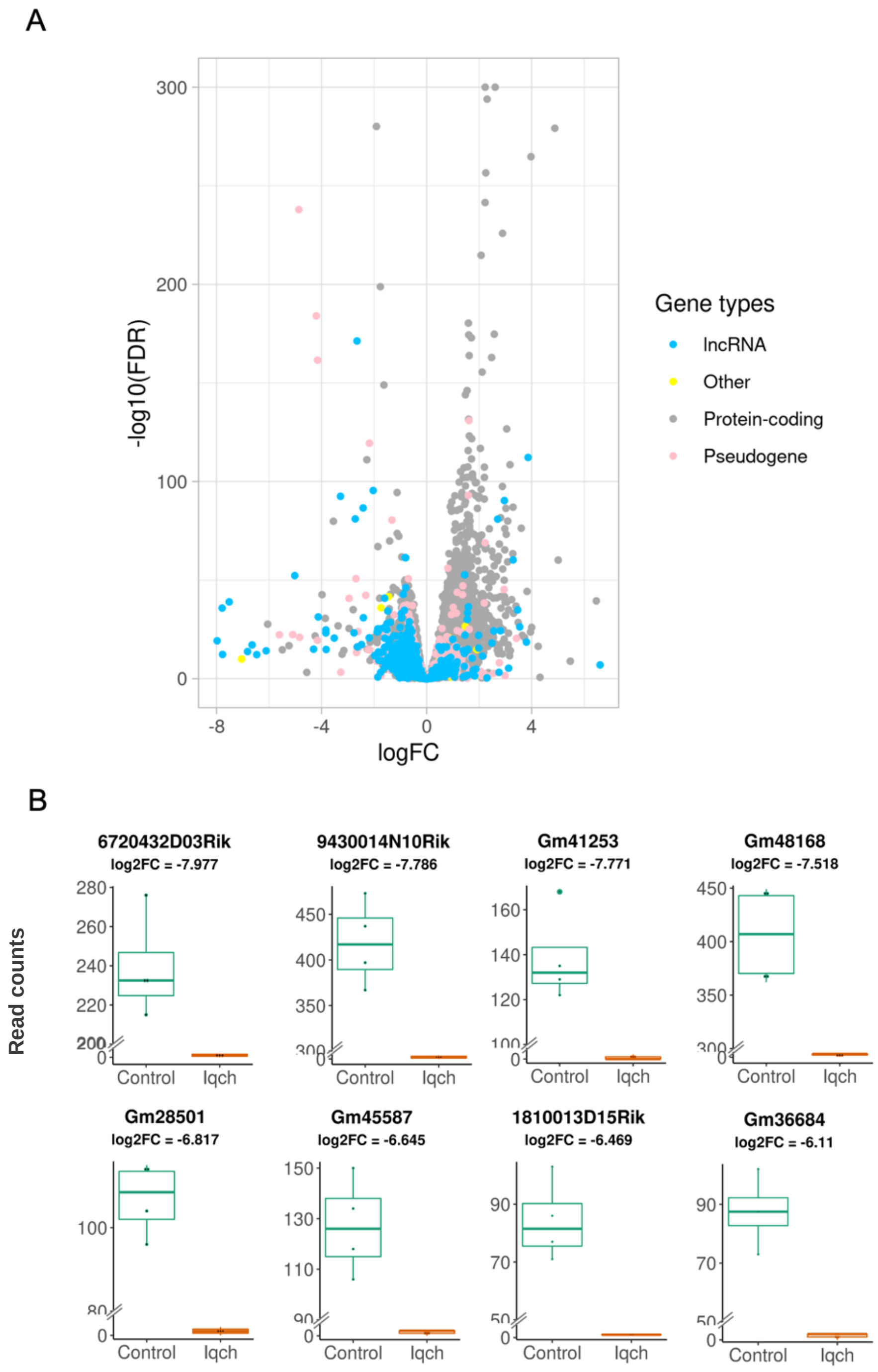

Fig. S10. **RNAseq results showing that lncRNAs are preferentially down regulated**. (**A**) Volcano plot of the differentially expressed genes in testis of control and *Iqch*^{mu} mice showing gene types and (**B**) boxplots of the 8 DE lncRNAs with highest fold change.

Development • Supplementary information

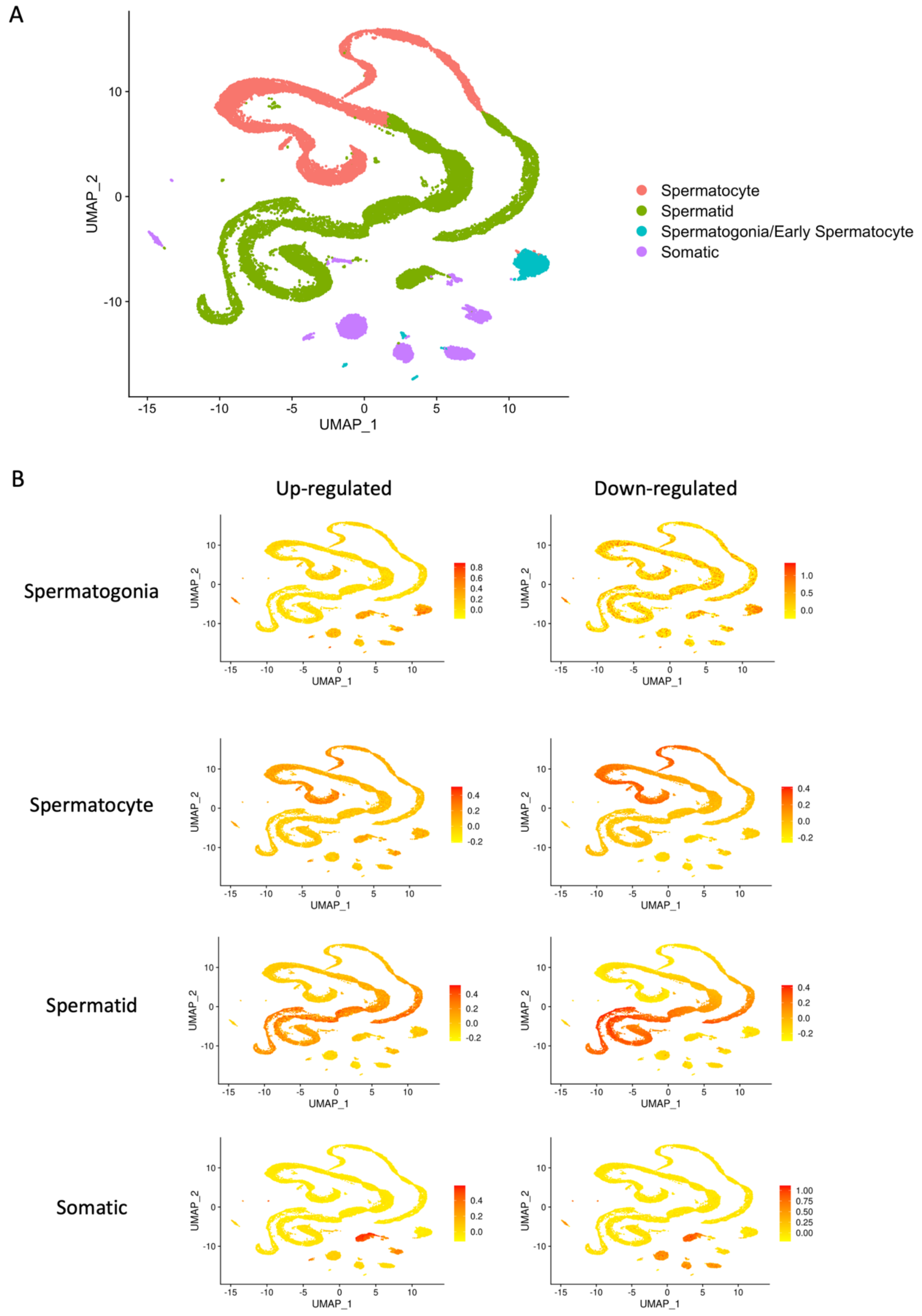

Fig. S11. **Single-cell analysis of ~35,000 testis cells using the sequencing data of the study of Green et al., (2018)**. (**A)** UMAP (Uniform Manifold Approximation and Projection for Dimension Reduction) plot with 4 clusters of the major cell types present in the seminiferous tubules. The different cell types are shown in different colors. (**B**) UMAP plot showing the log-scaled expression level in the Green et al., (2018) cells of the differentially expressed genes obtained for the *Iqch*^{mu} versus controls.

Development • Supplementary information

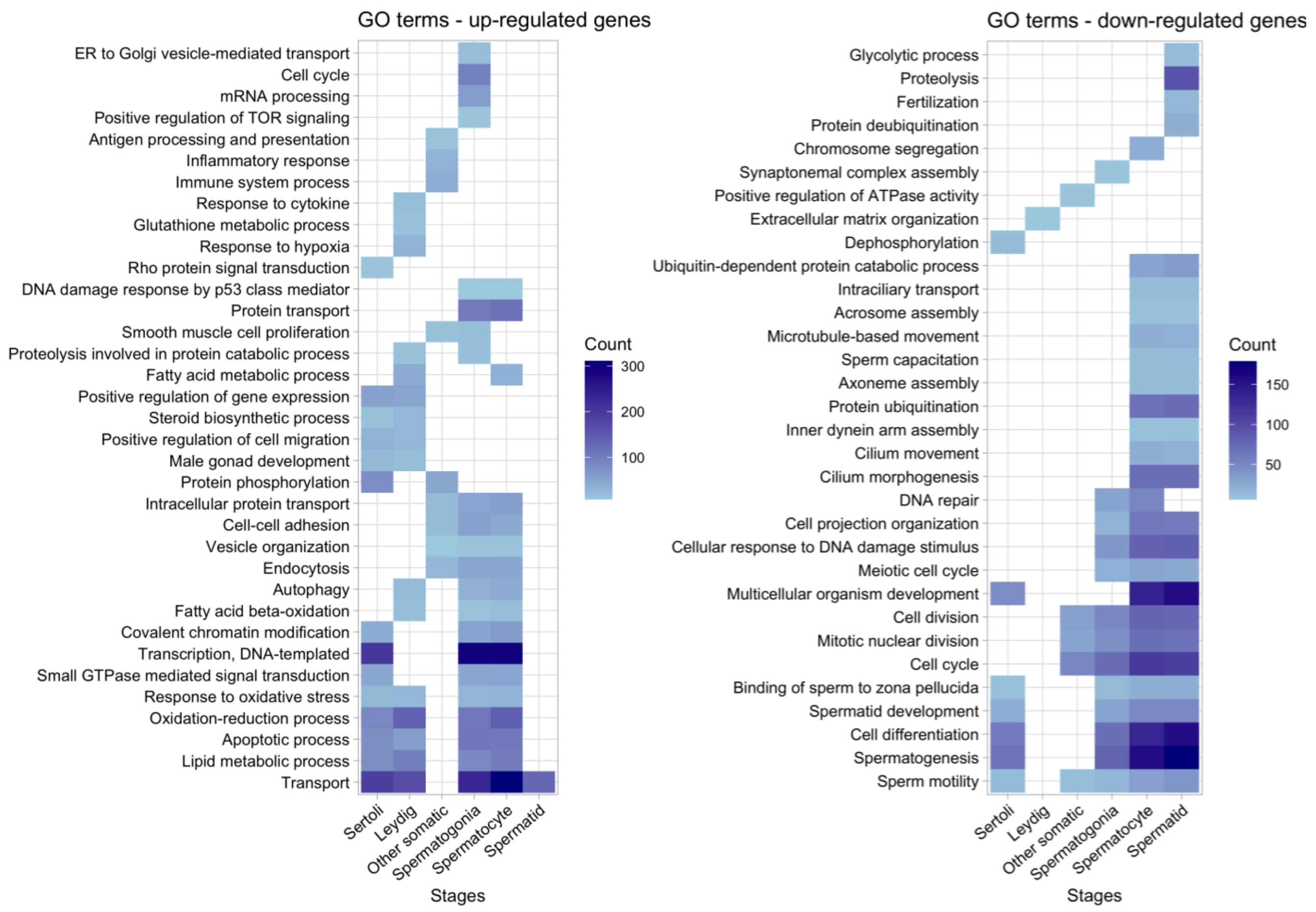

Fig. S12. Overrepresentation analysis of GO terms biological processes (GO-BP). Heatmaps that summarize the GO-BP terms in which the (A) upregulated and (B) downregulated genes of the different testis cell types are involved, with a color gradient showing the gene count.

Development • Supplementary information

A

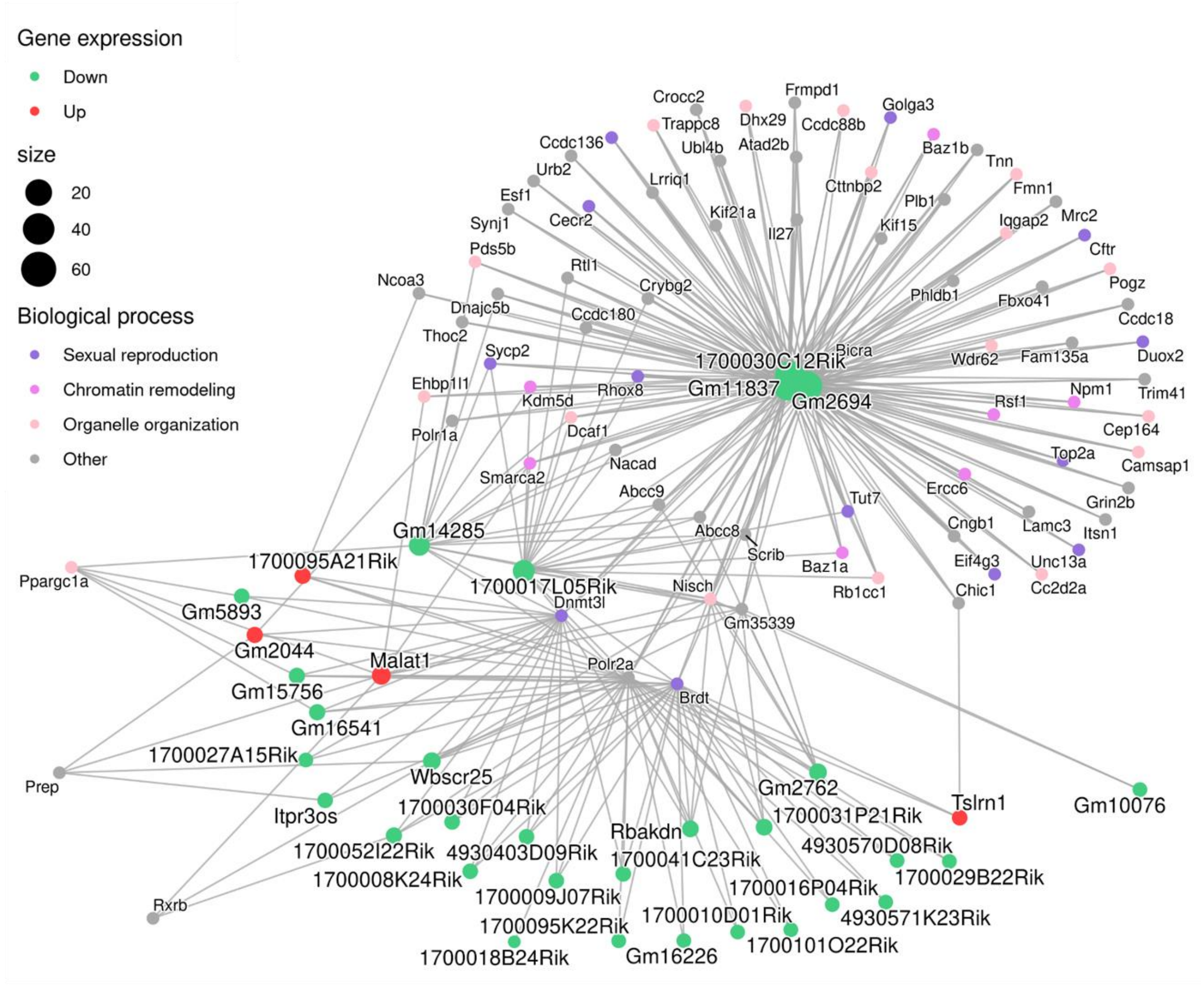

B

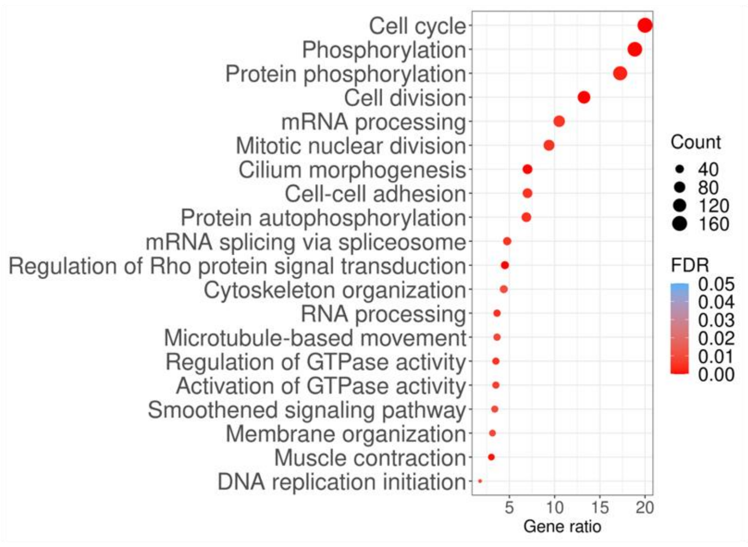

Fig. S13. Protein interactions of lncRNAs. A. Interaction network of the common differentially expressed lncRNAs, among germ cells and testicular somatic cells, with proteins. **B.** Dotplot showing the GO terms enriched in the target proteins that interact with the down-regulated lncRNAs of spermatids

Development • Supplementary information

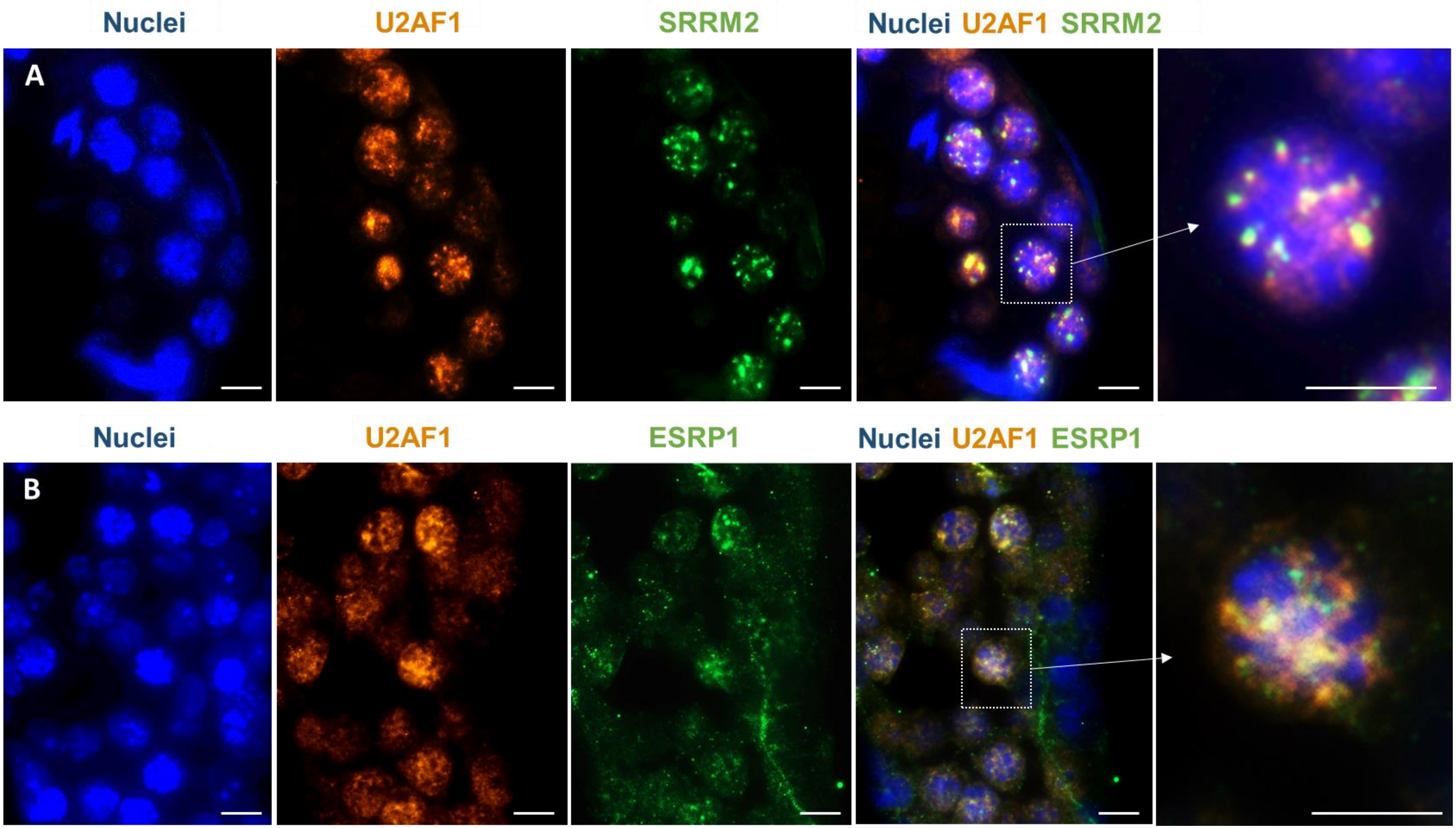

Fig. S14. Co-localization in WT testicular cells of SRRM2 and ESRP1 with the spliceosome and nuclear speckle marker U2AF1. (**A**) Representative section of adult mouse testicles immunostained with U2AF1 (orange), SRRM2 (green), and merged. On the right enlarged pictures of boxed areas, it is shown that U2AF1 spots co-localize with SRRM2 ots (yelow). (**B**) Representative section of adult mouse testicles immunostained with U2AF1 (orange), ESRP1 marks (green), and merged. On the right enlarged pictures of boxed areas, it is shown that U2AF1 spots co-localize with ESRP1 (yelow). Scale bar: 10 μm.

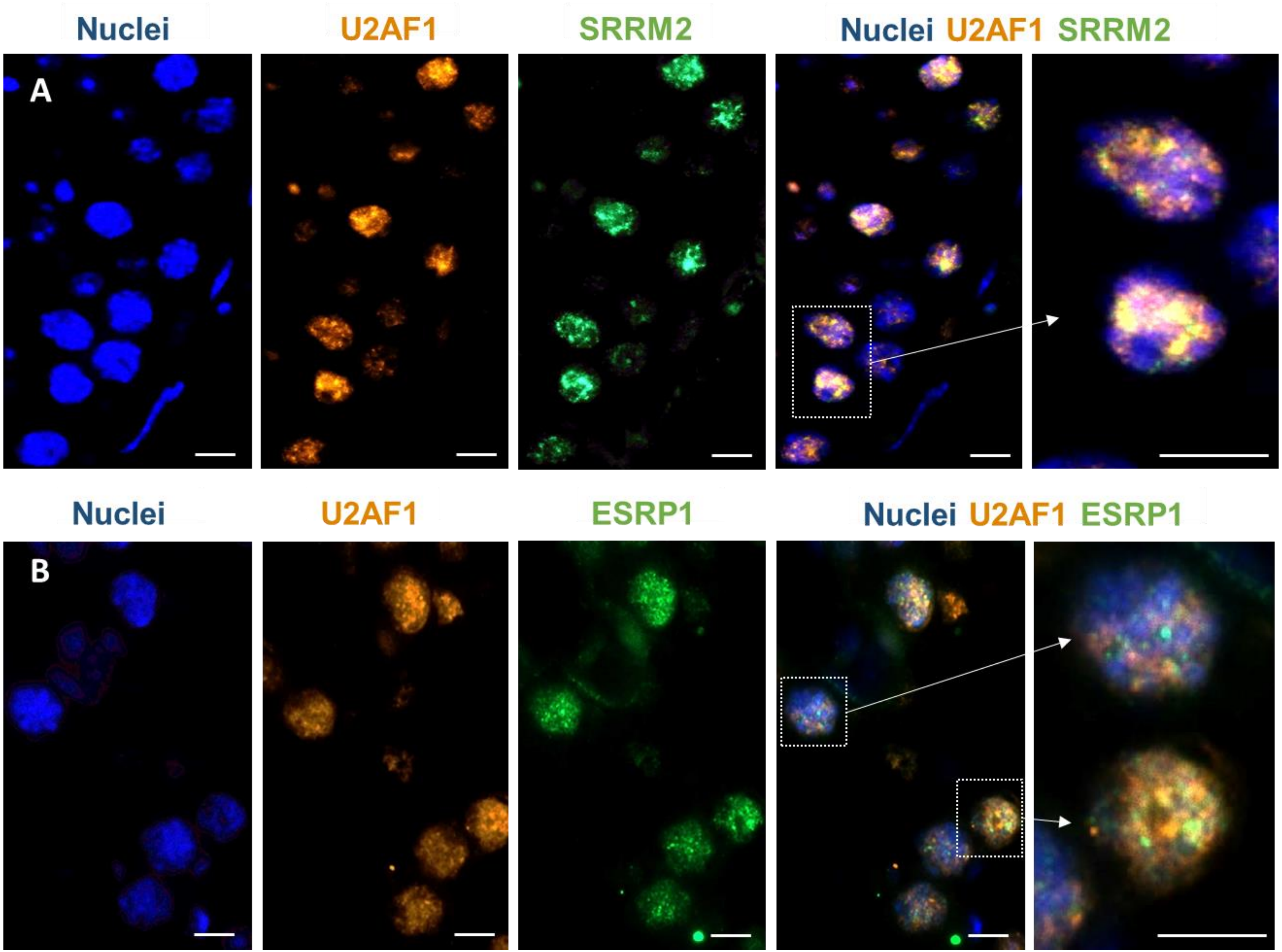

Fig. S15. Co-localization in *Iqch^mu* testicular cells of SRRM2 and ESRP1 with the spliceosome and nuclear speckle marker U2AF1. (**A**) Representative section of adult mouse testicles immunostained with U2AF1 (orange), SRRM2 (green), and merged. On the right enlarged pictures of boxed areas, it is shown that U2AF1 spots co-localize with SRRM2 ots (yelow). (**B**) Representative section of adult mouse testicles immunostained with U2AF1 (orange), ESRP1 marks (green), and merged. On the right enlarged pictures of boxed areas, it is shown that U2AF1 spots does not localize with ESRP1 (green and orange marks). Scale bar: 10 µm..

Development • Supplementary information

Annotation Cluster 1	Enrichment Score: 31.93		Count	P_Value	Benjamini
UP_KEYWORDS	RNA-binding	RT	34	6.8E-40	6.7E-38
GOTERM_MF_DIRECT	RNA binding	RT	34	1.9E-32	3.0E-30
GOTERM_MF_DIRECT	poly(A) RNA binding	RT	37	6.7E-32	5.3E-30
GOTERM_CC_DIRECT	intracellular ribonucleoprotein complex	RT	23	2.2E-26	2.8E-24
**Annotation Cluster 2**	**Enrichment Score: 21.87**		**Count**	**P_Value**	**Benjamini**
INTERPRO	RNA recognition motif domain	RT	22	9.3E-28	1.0E-25
INTERPRO	Nucleotide-binding, alpha-beta plait	RT	22	2.5E-26	1.4E-24
SMART	RRM	RT	22	1.1E-24	3.0E-23
GOTERM_MF_DIRECT	nucleic acid binding	RT	31	6.0E-22	3.2E-20
UP_SEQ_FEATURE	domain:RRM 1	RT	16	1.6E-21	1.8E-19
UP_SEQ_FEATURE	domain:RRM 2	RT	16	1.6E-21	1.8E-19
GOTERM_MF_DIRECT	nucleotide binding	RT	29	2.3E-14	9.2E-13
**Annotation Cluster 3**	**Enrichment Score: 12.49**		**Count**	**P_Value**	**Benjamini**
GOTERM_BP_DIRECT	mRNA processing	RT	18	4.5E-18	1.4E-15
UP_KEYWORDS	Spliceosome	RT	13	2.6E-17	5.2E-16
UP_KEYWORDS	mRNA processing	RT	16	1.3E-16	1.5E-15
UP_KEYWORDS	mRNA splicing	RT	15	1.3E-16	1.5E-15
UP_KEYWORDS	Ribonucleoprotein	RT	16	1.4E-16	1.5E-15
GOTERM_CC_DIRECT	catalytic step 2 spliceosome	RT	12	8.3E-16	5.4E-14
GOTERM_CC_DIRECT	spliceosomal complex	RT	12	4.3E-14	1.4E-12
GOTERM_BP_DIRECT	RNA splicing	RT	14	5.2E-14	8.1E-12
UP_KEYWORDS	Viral nucleoprotein	RT	7	6.9E-12	5.7E-11
KEGG_PATHWAY	Spliceosome	RT	11	1.1E-11	8.3E-10
GOTERM_CC_DIRECT	viral nucleocapsid	RT	7	1.6E-11	3.5E-10
UP_KEYWORDS	Virion	RT	7	2.7E-11	2.1E-10
INTERPRO	Nuclear factor hnRNPA1	RT	3	1.8E-5	4.1E-4
UP_SEQ_FEATURE	region of interest:RNA-binding RGG-box	RT	3	4.3E-4	1.1E-2
**Annotation Cluster 4**	**Enrichment Score: 6.08**		**Count**	**P_Value**	**Benjamini**
UP_SEQ_FEATURE	domain:DRBM 1	RT	5	2.0E-8	1.1E-6
UP_SEQ_FEATURE	domain:DRBM 2	RT	5	2.0E-8	1.1E-6
SMART	DSRM	RT	5	6.1E-7	8.5E-6
INTERPRO	Double-stranded RNA-binding-like domain	RT	5	1.5E-6	5.5E-5
GOTERM_MF_DIRECT	double-stranded RNA binding	RT	4	1.1E-3	1.3E-2
**Annotation Cluster 5**	**Enrichment Score: 4.48**		**Count**	**P_Value**	**Benjamini**
GOTERM_MF_DIRECT	mRNA 3'-UTR binding	RT	5	1.7E-5	3.3E-4
GOTERM_BP_DIRECT	negative regulation of mRNA splicing, via spliceosome	RT	4	4.1E-5	2.2E-3
GOTERM_MF_DIRECT	mRNA CDS binding	RT	3	5.0E-5	7.9E-4
**Annotation Cluster 6**	**Enrichment Score: 3.1**		**Count**	**P_Value**	**Benjamini**
UP_KEYWORDS	Biological rhythms	RT	5	1.9E-4	1.1E-3
GOTERM_BP_DIRECT	rhythmic process	RT	5	4.0E-4	1.4E-2
GOTERM_CC_DIRECT	extracellular matrix	RT	5	7.1E-3	6.1E-2
**Annotation Cluster 7**	**Enrichment Score: 2.65**		**Count**	**P_Value**	**Benjamini**
INTERPRO	DNA/RNA helicase, DEAD/DEAH box type, N-terminal	RT	5	2.6E-5	4.8E-4
GOTERM_MF_DIRECT	ATP-dependent RNA helicase activity	RT	5	3.4E-5	5.9E-4
UP_SEQ_FEATURE	domain:Helicase C-terminal	RT	5	1.1E-4	3.5E-3

Fig. S16: **Summary of functional annotation clustering analysis (DAVID) of the 54 interacting proteins for IQCH from the Pathway Commons Protein-Protein Interactions dataset**. The clustering of ontological terms found in the dataset are related to 1) RNA binding, poly(A) RNA binding, and ribonucleoprotein complex; 2) RNA recognition motif domain and nucleic acid binding; 3) mRNA processing, spliceosome, and mRNA splicing; 4) Double-stranded RNA-binding; 5) mRNA 3´-UTR binding. UP_KEYWORDS, UniProt Keywords annotation of genes; UP_SEQ_FEATURE, UniProt sequence feature annotation of genes; GOTERM_BP_DIRECT, Gene ontology biological process annotation; GOTERM_CC_DIRECT, Gene ontology cellular component annotation; GOTERM_MF_DIRECT, Gene ontology molecular functions annotation; INTERPRO, Classification of protein families; SMART, Simple modular architecture research tool.

Development • Supplementary information

Table S1. RNA-seq data analysis and differential gene expression analysis. A) Summary of RNA-seq data. B) Differential gene expression analysis by edgeR and DEseq2 (FDR<0.01).

Click here to download Table S1

Table S2. GO biological process terms significantly overrepresented (FDR<0.05) in upregulated and downregulated genes.

Click here to download Table S2

Table S3. Classification of DEGs according to their expression in the different testis cell types (spermatogonia, spermatocyte, spermatid, sertoli, leydig, other somatic cells and sperm). A) Summary of DEGs detected (adjusted p-value<001 or with FC>2) according the different cell types in which this genes were classified. B) Summary of DEGs according to cell types and the main gene types

Click here to download Table S3

Table S4. GO biological process terms significantly over-represented (FDR<0.05) in the DEGs belonging to the different testis cell types (in both upregulated and downregulated DEGs).

Click here to download Table S4

Table S5. Interactions of lncRNA with proteins and mRNAs (RNAInter database).

Click here to download Table S5

Table S6. Differential splicing events obtained by rMATS.

Click here to download Table S6

Table S7. Differential isoform analysis, including both differential expression (DTE) and differential usage (DTU) approaches.

Click here to download Table S7

Table S8. Proteins that interact with IQCH identified by the Pathway Commons Protein-Protein Interactions Dataset from Harmonizone.

Click here to download Table S8

Table S9. Primers used in this study.

Click here to download Table S9

Development • Supplementary information

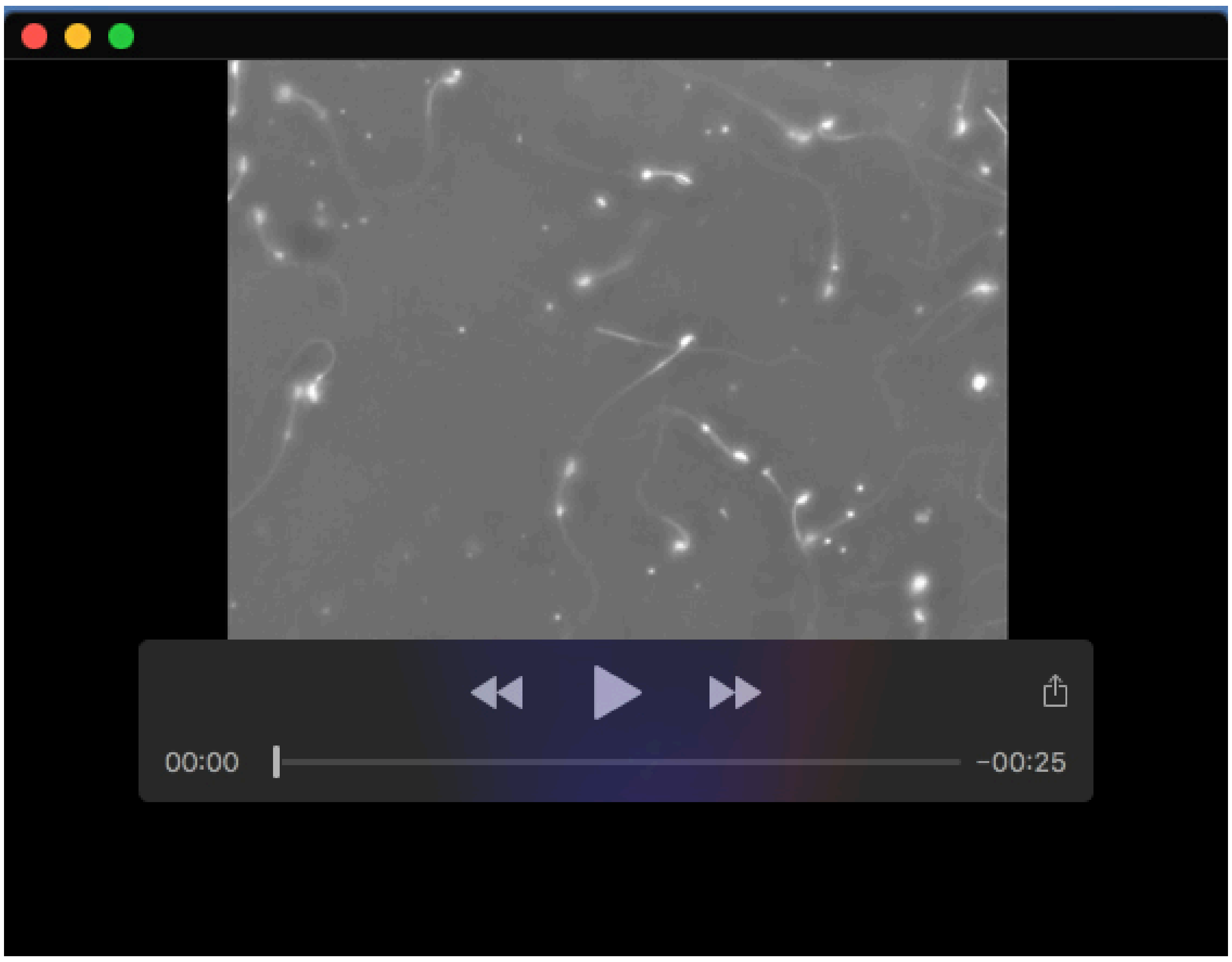

Movie 1. Lower motility of *Iqch*^{mu} spermatozoa. Sperm motility was observed using a high-speed camera (200 frames/second). Cauda epididymis spermatozoa were incubated in TYH medium and motility was recorded after 10 min (non-capacitating) of incubation. The movies are played at 20 frames/second (1/10 speed). WT sperm,

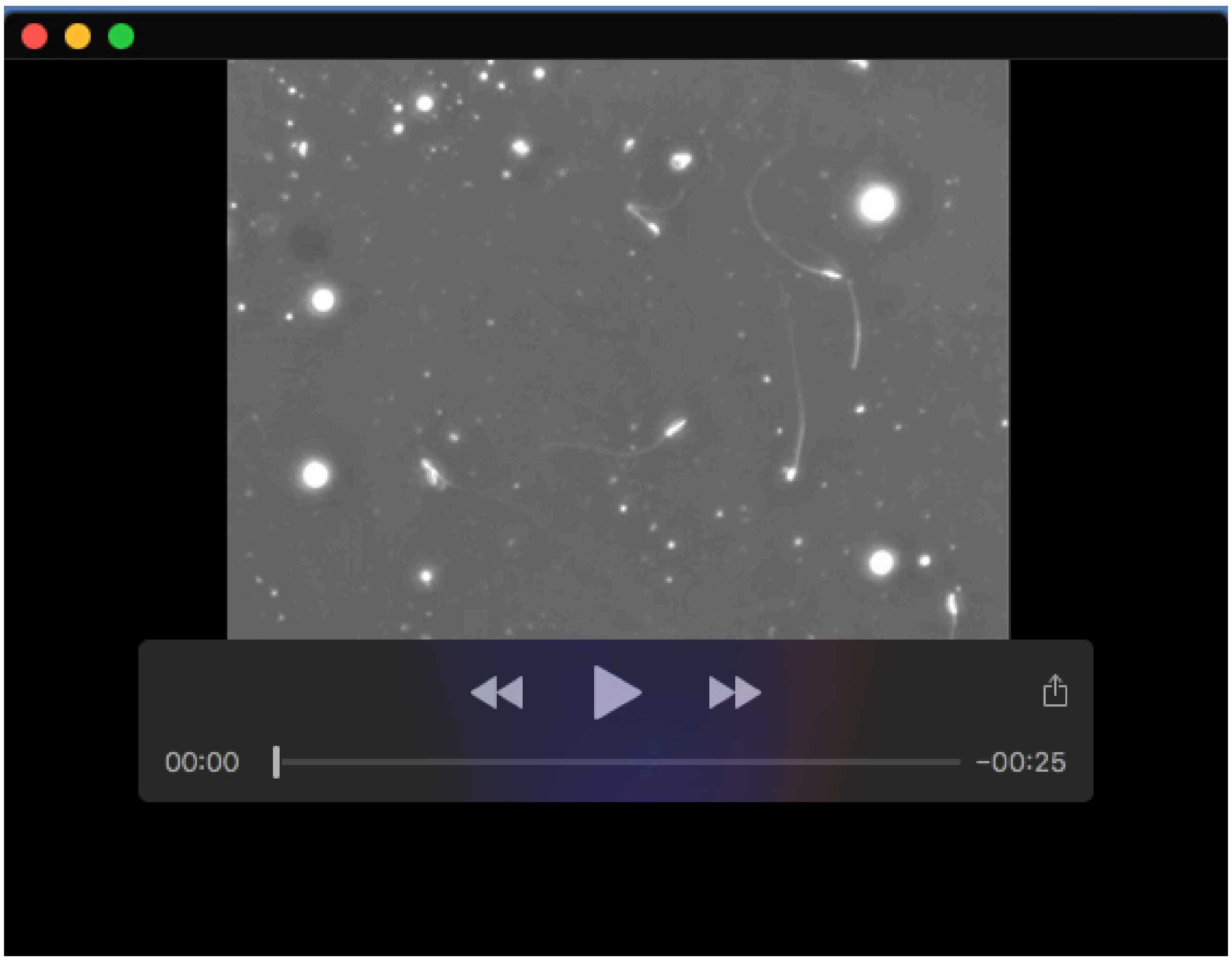

Movie 2. Lower motility of *Iqch*^{mu} spermatozoa. Sperm motility was observed using a high-speed camera (200 frames/second). Cauda epididymis spermatozoa were incubated in TYH medium and motility was recorded after 10 min (non-capacitating) of incubation. The movies are played at 20 frames/second (1/10 speed). *Iqch*^{mu} sperm.
